# Prognostic modeling of oral cancer by gene profiles and clinicopathological co-variables

**DOI:** 10.18632/oncotarget.19576

**Published:** 2017-07-26

**Authors:** Steven W. Mes, Dennis te Beest, Tito Poli, Silvia Rossi, Kathrin Scheckenbach, Wessel N. van Wieringen, Arjen Brink, Nicoletta Bertani, Davide Lanfranco, Enrico M. Silini, Paul J. van Diest, Elisabeth Bloemena, C. René Leemans, Mark A. van de Wiel, Ruud H. Brakenhoff

**Affiliations:** ^1^ Department of Otolaryngology – Head and Neck Surgery, VU University Medical Center, Amsterdam, The Netherlands; ^2^ Department of Epidemiology and Biostatistics, VU University Medical Center, Amsterdam, The Netherlands; ^3^ Unit of Maxillo-Facial Surgery, Department of Biomedical, Biotechnological and Translational Sciences (S.Bi.Bi.T.), University of Parma, Parma, Italy; ^4^ COMT & Department of Life Science, University of Parma, Parma, Italy; ^5^ Department of Otorhinolaryngology, Head and Neck Surgery, Medical Faculty Heinrich Heine University, Düsseldorf, Germany; ^6^ Department of Mathematics, VU University Amsterdam, Amsterdam, The Netherlands; ^7^ Department of Pathology and Laboratory Medicine, University of Parma, Parma, Italy; ^8^ Department of Pathology, University Medical Center Utrecht, Utrecht, The Netherlands; ^9^ Department of Pathology, VU University Medical Center, Amsterdam, The Netherlands; ^10^ Department of Maxillofacial Surgery/Oral Pathology, Academic Medical Centre for Dentistry, Amsterdam, The Netherlands

**Keywords:** head and neck cancer, oral cancer, lymph node metastasis, prognostic modeling, expression profiling

## Abstract

Accurate staging and outcome prediction is a major problem in clinical management of oral cancer patients, hampering high precision treatment and adjuvant therapy planning. Here, we have built and validated multivariable models that integrate gene signatures with clinical and pathological variables to improve staging and survival prediction of patients with oral squamous cell carcinoma (OSCC). Gene expression profiles from 249 human papillomavirus (HPV)-negative OSCCs were explored to identify a 22-gene lymph node metastasis signature (LNMsig) and a 40-gene overall survival signature (OSsig). To facilitate future clinical implementation and increase performance, these signatures were transferred to quantitative polymerase chain reaction (qPCR) assays and validated in an independent cohort of 125 HPV-negative tumors. When applied in the clinically relevant subgroup of early-stage (cT1-2N0) OSCC, the LNMsig could prevent overtreatment in two-third of the patients. Additionally, the integration of RT-qPCR gene signatures with clinical and pathological variables provided accurate prognostic models for oral cancer, strongly outperforming TNM. Finally, the OSsig gene signature identified a subpopulation of patients, currently considered at low-risk for disease-related survival, who showed an unexpected poor prognosis. These well-validated models will assist in personalizing primary treatment with respect to neck dissection and adjuvant therapies.

## INTRODUCTION

Head and neck squamous cell carcinoma (HNSCC) is the 7^th^ most common tumor in the world [1]. HNSCC originates in the mucosal linings of the oral cavity, oropharynx, hypopharynx and larynx. The majority of patients (30-40%) present with oral squamous cell carcinoma (OSCC) [2]. Classical risk factors for HNSCC are tobacco use and alcohol consumption. Additionally, human papillomavirus (HPV) infection became manifest as a cause during the last decade. The HPV-attributable fraction is highest in oropharyngeal squamous cell carcinoma (OPSCC), and varies from 20-90% depending on the geographical region [3]. Also oral cancers may arise from HPV infection, but the attributable fraction is lower, ranging from 0-6% [4]. OPSCCs caused by HPV infection are different at the molecular level [5] and have a highly favorable prognosis [6]. This different clinical behavior led to treatment de-intensifying trials to personalize treatment and a staging adaptation in the 8^th^ edition of the TNM Classification of Malignant Tumors of the Union for International Cancer Control (UICC) [7].

The 5-years overall survival for OSCC is 60%, but ranges from 10 to 80% depending on the extent of the tumor at diagnosis [8], as defined by the TNM stage. TNM staging is based on prognosis and employed for treatment planning in patients with OSCC [9], but is group-based and meets limitations for personalizing treatment of the individual patient.

OSCC is mainly treated by surgery with or without postoperative radiotherapy or chemoradiotherapy, and besides TNM stage, additional important prognostic features are derived from histopathological examination of the surgical specimen. For example, tumor-positive surgical margins (R+) and lymph node metastasis (LNM) with extracapsular spread (ECS) are classical treatment-decisive prognostic factors and indicators for postoperative chemoradiotherapy. Of note, histopathological examination of the specimen is only available for postoperative therapy decisions, and not for pre-treatment prediction of prognosis and treatment planning. Particularly for patients with a clinically N0 neck an important choice has to be made between elective treatment of the neck, with associated morbidity, or active surveillance with the risk of occult lymph node metastases that will become manifest during follow-up. Molecular profiling of tumor specimen may provide additional, objective information to improve current prognostication, and can even be performed on pretreatment biopsies to stage the neck.

Several prognostic models based on molecular profiles have been evaluated for HNSCC in general, or for OSCC specifically [10–13]. These models predicted survival of the studied populations, and added independent information to other established prognostic factors. However, none of these models has been introduced in clinical practice. Reasons are (1) insufficient clinical validation of the models, (2) the complexity and lack of reproducibility of the different profiling platforms [14], (3) heterogeneous study populations regarding HPV status and tumor subsite, (4) the high costs of transcriptomic profiling, and (5) the lack of compatibility with formalin-fixed paraffin-embedded (FFPE) tissue specimen. Translation of expression profiles to quantitative real-time polymerase chain reaction (qPCR) platforms using selected gene panels may overcome most of these disadvantages.

Another argument holds true for expression profiles associated with the clinically N0 neck. Previously, an expression profile has been identified and appropriately validated in a multicenter trial [15–17]. The signature remained accurate with negative predictive values (NPV) of 88% to 90% in the clinically relevant subgroup. However, the sentinel node biopsy is a competing diagnostic modality in this patient group with an even higher NPV of 95% [18]. Notwithstanding, sentinel node biopsy has not been introduced widely, has a poor performance for floor of mouth tumors, and has the obvious disadvantage that it remains a surgical procedure with radioactive tracers, whereas for gene expression analysis only a biopsy is required. Particularly, switching to RT-qPCR analysis of a thoroughly selected gene panel may further enhance the predictive power of the gene signature because of the large dynamic range of RT-qPCR.

We therefore aimed to identify and test gene expression signatures to address these important challenges in head and neck oncology: prediction of lymph node metastasis (LNM) and overall survival (OS). First, signatures of informative genes were selected from gene expression data by regression methods. Next, a limited number of genes were selected for platform transition to RT-qPCR assays, and the prognostic power was validated using an independent cohort of surgically-treated HPV-negative OSCC patients. The molecular data were further combined with clinical and pathology data to provide the most accurate models for clinical practice to predict nodal metastatic disease and prognosis.

## RESULTS

Microarray data from two cohorts, 150 OSCC patients from The Netherlands (Array Cohort 1, AC1) and 99 OSCC patients from Italy (Array Cohort 2, AC2), were used to identify genes related to LNM and OS (Table 1). LNM was present in 60% of AC1 patients and 49.5% of AC2 patients. In AC1, the median overall follow-up time was 7.2 years (95% CI = 6.7 – 8.1). In AC2, the median overall follow-up time was 3.5 years (95% CI = 3.3 – 4.3).

**Table 1:** Characteristics of patients in the four study cohorts^a^

Characteristic	Array cohort 1	Array cohort 2	qPCR cohort	TCGA cohort	P^b^ value
	(n = 150)	(n = 99)	(n = 125)	(n = 160)	
Age, mean (SD)	62 (10.7)	66 (10.3)	63 (12.6)	62 (13.6)	P=0.06
Gender					
Male (%)	90 (60.0)	54 (54.5)	72 (57.6)	105 (65.6)	P=0.30
Female (%)	60 (40.0)	45 (45.5)	53 (42.2)	55 (34.4)	
Smoking (PY)					
0-10 (%)	36 (24.0)	51 (51.5)	41 (32.8)	47 (29.4)	P<0.001
11-24 (%)	19 (12.7)	10 (10.1)	13 (10.4)	13 (8.1)	
>24 (%)	95 (63.3)	38 (38.4)	71 (56.8)	60 (37.5)	
Unknown (%)	-	-	-	40 (25.0)	
Subsite					
Oral tongue (%)	53 (35.3)	41 (41.4)	48 (38.4)	-	P=0.62
Other oral cavity (%)	97 (64.7)	58 (58.6)	77 (61.6)	-	
TNM stage					
I (%)	18 (12.0)	22 (22.2)	16 (12.8)	10 (6.3)	P=0.02
II (%)	22 (14.7)	12 (12.1)	27 (21.6)	32 (20.0)	
III (%)	31 (20.7)	21 (21.2)	26 (20.8)	25 (15.6)	
IV (%)	79 (52.7)	44 (44.4)	56 (44.8)	82 (51.3)	
Unknown (%)	-	-	-	11 (6.9)	
N-stage					
Negative (%)	60 (40)	48 (48.5)	61 (48.8)	57 (35.6)	P=0.35
Positive (%)	90 (60)	49 (49.5)	64 (51.2)	76 (47.5)	
Unknown	-	2 (2.0)	-	27 (16.9)	
pCompVar^c^					
Negative (%)	-	-	79 (63.2)	-	
Positive (%)	-	-	38 (30.4)	-	
Unknown (%)	-	-	8 (6.4)	-	

pCompVar, pathological composite variable; PY, packyears; SD, standard deviation.

a. Percentages may not total 100 because of rounding.

b. P values were calculated with the use of One-Way ANOVA for continuous variables and χ^2^ test for categorical variables.

c. Scored positive if extracapsular spread or positive resection margins or >1 lymph node metastasis was present.

### Identification of genes for prediction of lymph node metastasis and survival in OSCC

**Figure 1:**
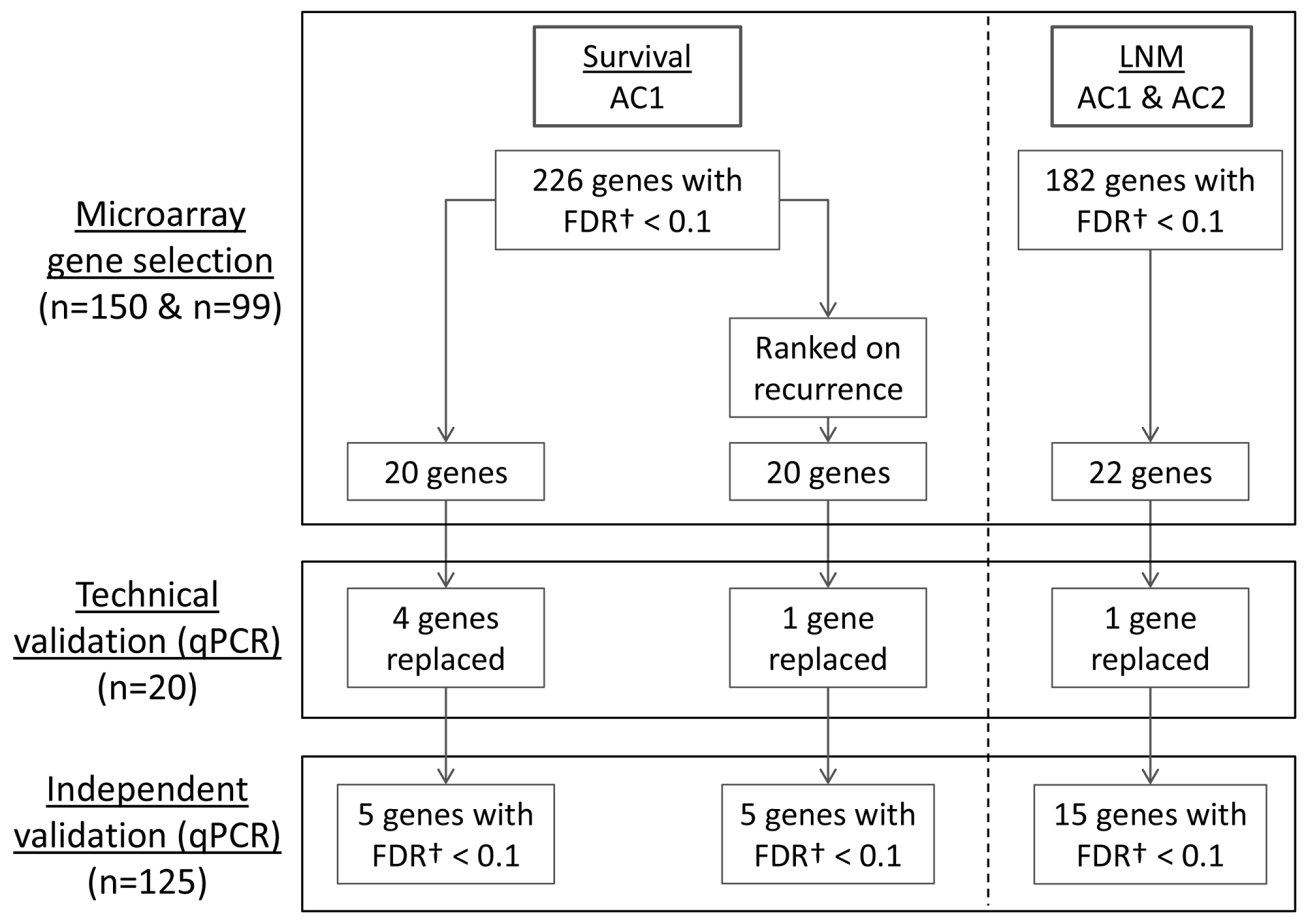
Schematic representation of the different phases of the study Two microarray cohorts (Array Cohort 1 (AC1), n=150; Array Cohort 2 (AC2), n=99) were explored by univariable and multivariable gene selection to identify a 22-gene lymph node metastasis signature (LNMsig) and a 40-gene overall survival signature (OSsig). For the OSsig, 20 genes were selected that were predictive for OS, and 20 additional genes were selected after the genes were ranked on their predictive value for recurrent disease to account for disease-specific death. For LNM prediction, a previously validated multigene microarray signature(15–17) was used as preselection. Subsequently, our signatures were transferred to RT-qPCR assays and correlated to the microarray data in 20 cases (technical validation). After this technical validation, 6 genes with poor correlation coefficients were replaced by the second best genes from the initial microarray analyses. Finally, the definitive signatures were validated on an independent cohort of 125 tumors (independent validation). †Univariable p-values were corrected for multiple testing using the Benjamini-Hochberg FDR procedure. AC1, Array Cohort 1; AC2, Array Cohort 2; FDR, false discovery rate; LNM, lymph node metastasis; qPCR, quantitative polymerase chain reaction.

The gene selection strategy is summarized in Figure 1 and described in detail in the Supplementary Materials. In short, the previously published LNM gene profile [15, 17] was evaluated to predict N-stage in AC1 and AC2. Using the global test with pathological N-stage as outcome, these genes had a p-value of 9.3E-06 and 9.9E-03 in AC1 and AC2, respectively. Combined univariable analysis identified 221 significant genes (FDR<0.1, Supplementary Table 1). From these genes, 22 genes were selected for RT-qPCR validation based on their ranking in univariable and multivariable analysis.

For survival, a similar gene pre-selection strategy was hampered by the lack of thoroughly validated prognostic gene signatures in the public domain. We therefore included other techniques to reduce the dimensions of the data, but also explored all genes to ensure that important prognostic genes were not missed. We only used AC1 for gene selections, as AC2 did not pass the global test due to the shorter follow-up time (global test p-values AC1: 7.8E-3 and AC2: 0.73). Univariable analysis of all genes identified 226 (out of 37,662) significant genes in AC1 (FDR<0.1, Supplementary Table 1). Next, 20 genes were selected by univariable and multivariable analyses for survival, and 20 additional genes were selected after ranking the genes on their predictive value for recurrent disease to account for disease-specific death (see Figure 1). Two genes overlapped between the 40 survival genes and the 22 LNM genes (Supplementary Figure 1), rendering an overall signature of 60 target genes for technical and independent RT-qPCR validation (Supplementary Table 2).

### Technical RT-qPCR validation of identified genes

First, the 60 target genes were technically validated in a subset of 20 cases from AC2 to evaluate the platform transition. For these 20 cases, correlation coefficients were calculated between microarray and corresponding RT-qPCR data (Supplementary Table 3). In total, 52 of 60 genes validated well, as they showed a good correlation between microarray and RT-qPCR data (mean r=0.64, SD=0.26). The remaining 8 genes correlated poorly, showing a correlation coefficient >1 SD below the mean. Cox regression nonetheless indicated that two of these eight genes did correlate with survival, i.e. EIF5 (P=0.011) and ATP6V0A1 (P=0.057), and these were therefore kept in the panel. The remaining 6 genes were replaced by the second best genes from the initial microarray analyses (Supplementary Table 1), and subsequently analyzed.

### Independent RT-qPCR validation of selected genes

The RT-qPCR validation cohort consisted of 125 OSCC cases that were independent from both microarray cohorts. In this validation cohort, nodal metastasis was detected in 51.2% of patients, and the median overall follow-up time was 5.1 years (95% CI = 4.4 – 6.3) (Table 1). The selected genes were run on customized microfluidic RT-qPCR cards, and the results were tested by univariable analyses and corrected for multiple testing. From the LNMsig 15 of 22 genes had an FDR<0.1 (Supplementary Table 4). From the OSsig 10 of 40 genes had an FDR<0.1 for OS, seven of which also significantly associated with disease-free survival (DFS). Thus, after correction for multiple testing, in total 25 of 60 genes selected from microarray datasets could be validated with RT-qPCR assays in an independent patient cohort.

### A gene expression-based model to predict lymph node metastasis in OSCC

**Figure 2:**
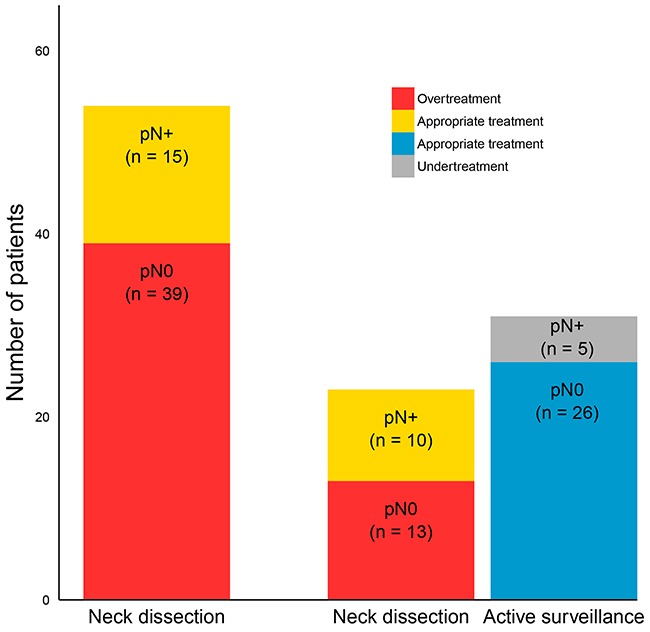
Incorporation of the LNMsig in a clinical decision model that was previously proposed for patients with clinically early stage (cT1-T2N0) oral squamous cell carcinoma (OSCC) At present, early-stage OSCCs are treated with an elective neck dissection (END, levels I-III or I-IV depending on location) in most centers. This would cause overtreatment in 39 patients (first bar, indicated in red). The clinical decision model recommends performing an END when the gene expression signature prediction is N+ or active surveillance when the prediction is N0. The hypothetical situation when using this decision model is represented in the second and third bar. Following the decision model, only 23 patients are directly treated with an elective neck dissection (second bar), overtreatment is restricted to 13 cases, and 26 patients receive appropriate treatment (third bar). The patients who are pN+ and receive an END are labeled as receiving appropriate treatment (indicated by yellow color).

The performance of the LNM predictive signature is summarized in Table 2; see Supplementary Table 4 for the estimates per gene. When all clinical stages of disease are considered, the AUC of this model was 0.69 (Table 2), with an NPV of 66% (Table 2). Next, we performed a subgroup analysis on the clinically relevant subset of tumors with clinical stages I and II (n=54), because these tumors qualify for transoral resection without treatment of the neck. In this subgroup, the AUC (0.66, Table 2) and the sensitivity of the LNMsig (67%, Table 2) were comparable with the performance statistics in all stages. The NPV, however, increased from 66% to 84% (Table 2). There were no clinical variables that correlated to LNM (data not shown) and data from histopathology is not available before surgery planning. Moreover, the fraction of occult lymph node metastasis was comparable in cT1 and cT2 tumors (i.e. 25% and 29% respectively). Previously, Van Hooff et al. [17] proposed a clinical decision model that recommends an elective neck dissection when the gene expression signature prediction indicates N+ or active surveillance when the prediction is N0, and estimated the benefit. Following this decision model, the LNMsig shows a similar benefit and could have prevented overtreatment in over 66% of the pN0 cases (72% or 24% overtreatment without or with the clinical decision model, respectively; see Figure 2).

**Table 2:** Performance metrics of gene signature in N-stage prediction

	qPCR validation, all	qPCR validation, cT1-2N0
	(n = 125)	(n = 54)
NPV (95% CI^a^)	66 (57.1-74.7)	84 (71.7-95.2)
TN	40	26
TN + FN	61	31
PPV (95% CI^a^)	67 (59.1-76.6)	43 (21.5-64.5)
TP	43	10
TP + FP	64	23
Sensitivity (95% CI^a^)	67 (42.3-83.5)	67 (29.6-93.2)
TP	43	10
TP + FN	64	15
Specificity (95% CI^a^)	66 (39.3-83.2)	67 (39.7-86.2)
TN	40	26
TN + FP	61	39
AUC (95% CI^a^)	0.69 (0.63-0.75)	0.66 (0.52-0.78)

AUC, Area Under the ROC Curve; CI, confidence interval; FN, false negative; FP, false positive; NPV, negative predictive value; PPV, positive predictive value; TN, true negative; TP, true positive.

a. Confidence intervals were assessed by bootstrapping.

### A gene expression-based prognostic model for OSCC with independent prognostic value

**Figure 3:**
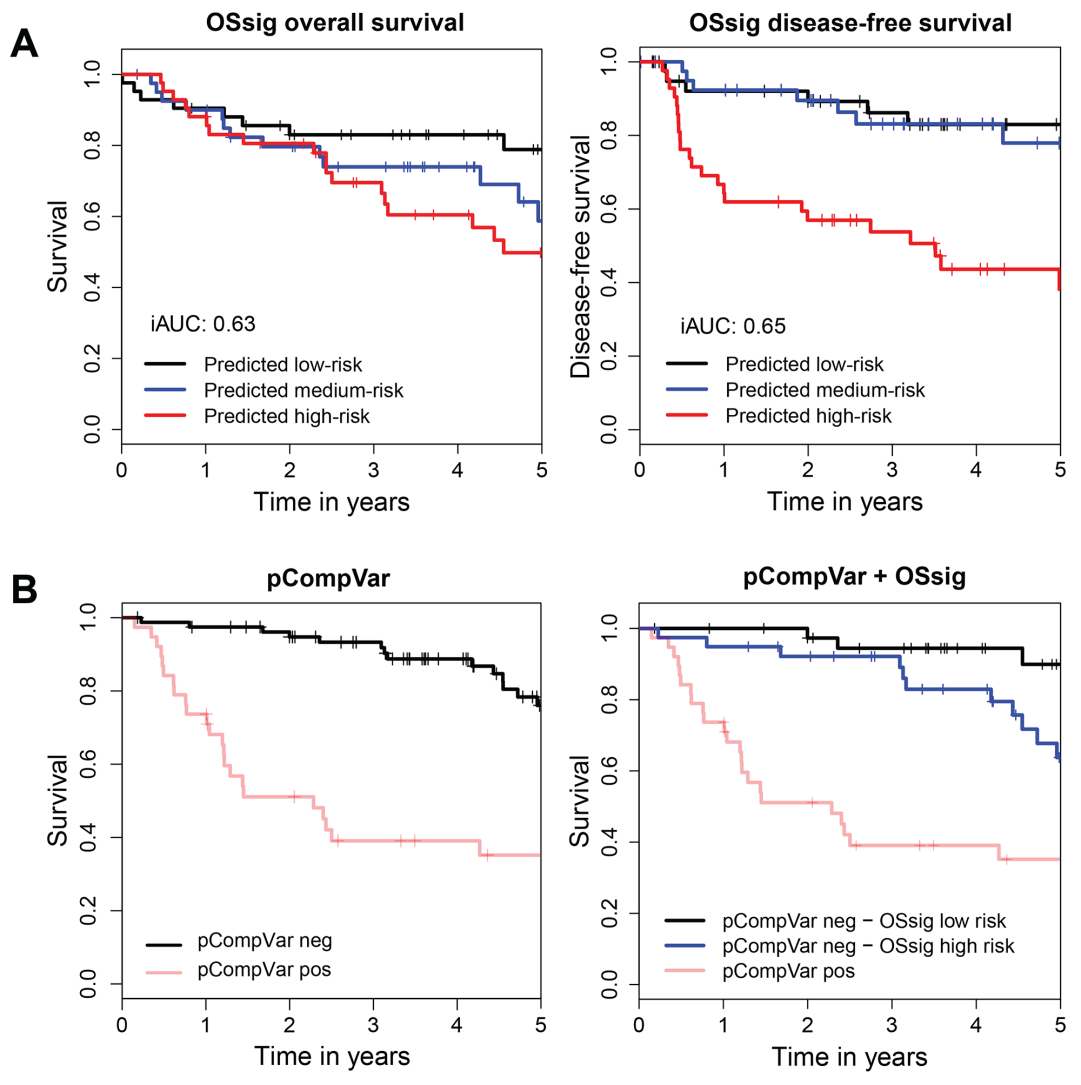
The overall survival signature (OSsig) predicts overall survival and disease-free survival, also in low-risk patients **(A)** Kaplan-Meier analysis of overall survival (left) and disease-free survival (right) with risk groups defined by tertile predicted hazards by the OSsig analyzed with qPCR in the independent validation cohort of 125 OSCC patients. We also considered threshold optimization for creating the three groups; resulting KM curves were very similar and are hence not displayed. **(B)** On the left, a Kaplan-Meier analysis is shown for overall survival in the independent validation group with risk groups defined by pCompVar, which is scored positive when during histopathological examination either extracapsular spread (ECS) or involved resection margins (R+) or >1 lymph node metastasis was identified. These are routinely used histopathological criteria for adjuvant treatment. On the right the result of a subgroup analysis is shown to improve the stratification of the pCompVar-*negative* patients (n=79). TNM-staging was not informative to stratify this group (data not shown), but the OSsig was able to identify a subgroup of patients (blue line) with relatively poor prognosis who might have benefited from adjuvant treatment (OS: iAUC=0.71; OSsig: P=0.01 (global test). The performances of all predicting models are listed in Table 3. Area under the curve was integrated over 5 year follow-up time. Tick marks on curves indicate censoring. iAUC, integrated Area Under the Curve; OSsig, Overall Survival signature; pCompVar, pathological composite variable.

The 40 survival genes significantly discriminated between high and low risk cases (OS: iAUC=0.63, P=1.6E-3 (global test), Table 3 and Figure 3A-left; DFS: iAUC=0.65, P=6.8E-3 (global test), Table 3 and Figure 3A-right; see Supplementary Table 4 for Ridge estimates per gene). In a clinical setting the genes should add prognostic information to established parameters. Hence, the gene signature was analyzed in context of clinical and histopathological data.

**Table 3:** Univariable and multivariable analysis of genomic, clinical, pathological and combined models in validation cohort

	Overall survival	P^c^ value	Disease free survival	P^c^ value
	iAUC^a^ (95% CI^b^)		iAUC^a^ (95% CI^b^)	
Unitype				
OSsig	0.63 (0.57-0.68)	0.002	0.65 (0.60-0.70)	0.007
Clinical	0.66 (0.59-0.73)		0.54 (0.49-0.61)	
pTNM	0.51 (0.47-0.57)		0.51 (0.47-0.57)	
pCompVar^d^	0.64 (0.56-0.71)		0.63 (0.56-0.71)	
Multitype				
Clinical+pTNM	0.66 (0.60-0.73)		0.53 (0.47-0.60)	
OSsig+clinical+pTNM	0.68 (0.64-0.73)	0.03	0.60 (0.55-0.64)	0.01
Clinical+pCompVar^d^	0.73 (0.67-0.80)		0.62 (0.54-0.70)	
OSsig+clinical+pCompVar^d^	0.74 (0.69-0.79)	0.02	0.68 (0.63-0.73)	0.01
pCompVar^d^ negative subgroup				
OSsig	0.71 (0.65-0.76)	0.01	0.65 (0.61-0.68)	0.28
Clinical	0.70 (0.61-0.79)		0.53 (0.43-0.68)	
OSsig+clinical	0.73 (0.68-0.78)	0.02	0.52 (0.46-0.65)	0.47

iAUC, integrated Area Under the Curve; OSsig, Overall Survival signature; pCompVar, pathological composite variable; pTNM, pathological TNM stage

a. Area under the curve was integrated over 5 year follow-up time.

b. CIs were assessed by bootstrapping on out-of-bag samples.

c. Significance of the OSsig was assessed with the global test^22,23^.

d. Scored positive if extracapsular spread or positive resection margins or >1 lymph node metastasis was present.

Several clinical factors were associated with OS, and none with DFS. A model was trained with the most important clinical factors for this dataset and pathological TNM-stage (pTNM). The clinical factors selected and included in the model were: age at diagnosis and smoking (i.e. packyears, PY), see Supplementary Table 5 for univariable p-values. The model with these two clinical factors and pTNM accurately predicted overall survival (iAUC=0.66, Table 3), but not DFS (iAUC=0.53, Table 3). Adding OSsig to this model improved the accuracy (OS: iAUC=0.68, OSSig: P=0.03 (global test), Table 3 and Supplementary Figure 2A). For DFS, a model based on the two clinical variables + pTNM and the OSsig gave an iAUC of 0.60. Note that this is lower than a model based on the OSsig only (iAUC 0.65).

Besides pTNM, other histopathological variables are important to decide on adjuvant treatment. In the Dutch guidelines, decisive criteria for adjuvant postoperative therapy are extracapsular spread (ECS), tumor-positive margins (R+) and multiple metastatic lymph nodes (>1 LNM). We created a composite variable (pCompVar) that was scored positive if ECS or R+ or >1 LNM was present. This composite variable was combined with clinical factors (i.e. age, PY) in a prognostic model (OS: iAUC=0.73, DFS: iAUC=0.62; see Table 3). The OSsig improved the accuracy of the model (OS: iAUC=0.74, OSsig: P=0.02 (global test), Table 3 and Supplementary Figure 2B; DFS: iAUC=0.68, OSsig: P=0.01 (global test), Table 3). DFS was most accurately predicted by a model that combined the OSsig and pCompVar, not including pTNM (iAUC=0.70; OSsig: P=5.6E-3 (global test)).

A subgroup analysis was performed with patients without criteria for postoperative radiotherapy, i.e. cases that were pCompVar-negative (n=79, Figure 3B-left). For these cases a multi-type prognostic model was built that included clinical factors (age and smoking) and the OSsig. The iAUC increased from 0.70 to 0.73 by adding the prognostic genes (Table 3 and Figure 3B-right). Predictive models for DFS were less accurate in this subgroup, although a predictive model with genes only showed some predictive power (iAUC=0.65, OSsig: P=0.27) (Supplementary Figure 3, Supplementary Table 3).

These findings show that the prognostic value of the OSsig adds to established clinical and pathological prognostic variables.

### External validation of LNMsig and OSsig with TCGA RNAseq data

For additional external validation, we used RNAseq data of HPV-negative OSCC tumors from the TCGA Network publication [19] (n=160, Table 1). The 22-gene LNMsig was significantly associated to pathological N-stage (P= 7.6E-06, global test). Moreover, the LNMsig could accurately classify the tumors with an AUC of 0.73 (95% CI = 0.67 to 0.78). The performance of the 40-gene OSsig was also significant (iAUC=0.59, P=0.02 (global test), Supplementary Figure 4). The OSsig was less informative since the average follow-up time for the 89 non-deceased cases was very short (2.2 years, SD = 2.35, Supplementary Figure 5A), and the baseline hazard was relatively high when compared to the RT-qPCR validation cohort (Supplementary Figure 5B).

## DISCUSSION

We identified prognostic gene expression signatures that are predictive of LNM and OS in OSCC by rigorous gene selection and validation. First, we selected 60 genes using microarray data, and these genes were validated in an independent cohort of OSCC patients by the use of RT-qPCR assays. Finally, we built 2 multivariable genomic models: a lymph node metastasis model (LNMsig) and overall survival model (OSsig) and confirmed the additive value of the gene signatures to existing and established variables.

The LNMsig with 22 genes predicted nodal metastatic disease with an NPV of 84% in clinical stages I and II. These diagnostic performance statistics are comparable to previous results using a 732-probe microarray signature [17]. However, the RT-qPCR approach facilitates clinical implementation considerably, because a comparable performance was achieved with less genes and a more user-friendly platform. A high NPV is necessary to identify patients who can be spared an elective neck dissection. Recent reports showed that the sentinel node biopsy (SNB), which is an alternative staging technique, is more accurate with an NPV of 95% [18] at comparable prevalence rates of LNM. The SNB, however, is an invasive surgical procedure with associated risks and costs, and with lower sensitivity in floor of mouth tumors [20–22]. Moreover, it has not been introduced widely. It has been suggested that a combination of an expression signature and SNB may be more accurate for staging of the clinically N0 neck [23].

The OSsig could be used to personalize treatment. By itself, the OSsig predicted overall survival with an iAUC of 0.63, which is already promising compared to the iAUC of 0.51 of standard pTNM. For prediction of DFS, the OSsig was even more valuable, particularly when combined with histopathology, as clinical variables were not informative for DFS. These data confirm the predictive value of the OSsig, but also indicate that integrating clinical, molecular and histopathological variables delivers most accurate predictive models.

The design of this study enabled the identification of robust associations in three ways. First, we used different gene expression platforms to cancel out platform-specific findings. Second, we studied homogeneous patient cohorts: only HPV-negative, surgically treated OSCCs were included. Finally, we considered patients from 3 European countries, thereby excluding the discovery of population-specific gene signatures.

Our findings may be limited by two factors. First, intra-tumor heterogeneity may cause differences in gene expression profiles within a tumor; although previous findings suggest that expression profiles seem stable in HNSCC [24]. Second, all cohorts investigated were retrospective. It should be mentioned, however, that retrospective data obtained in The Netherlands are generally accurate, because treatment and follow-up of HNSCC patients has been centralized to a few clinical centers and clinical management adheres to standardized national guidelines.

Our findings suggest at least two implications. First, the prognostic model may be used for treatment escalation in patients with tumors that do not fulfill the current criteria for postoperative radiotherapy, i.e. margin involvement, >1 metastatic lymph node or ECS. Second, a model that integrates clinical variables and the OSsig accurately predicts prognosis without the addition of histopathology. This model may specifically be important to predict survival in patients who are treated with primary radiotherapy or chemoradiotherapy, since histopathology is not available for these patients. These are important directions for future work. Since frozen material is not always available in these cases, future research should also include applications for FFPE tissue. Ultimately, prospective clinical trials will be required to determine whether the integrated risk models could guide clinical decision making and improve treatment results with respect to outcome and morbidity.

## MATERIALS AND METHODS

### Patients

Four independent cohorts of human papillomavirus (HPV)-negative OSCC patients were included (1) a cohort of 2 merged tumor gene expression profiles (array cohort 1, AC1) from the University Medical Center Utrecht (UMCU) and VU University Medical Center Amsterdam (VUmc); (2) a cohort of tumor gene expression profiles (array cohort 2, AC2) from the University Hospital Parma Medical Center (UHPMC); (3) an independent cohort of frozen tumor samples from VUmc, UHPMC and University Hospital Düsseldorf (UHD) for RT-qPCR gene expression profiling (qPCR cohort); and (4) an RNAseq dataset of OSCC tumors from The Cancer Genome Atlas (TCGA) Network [19]. Use of tissue from surgical specimen adhered to nation- and institution-specific procedures and guidelines. Informed consent was obtained of enrolled patients, when required. This study followed the Guidelines for the REporting of tumor MARKer Studies (REMARK) [25] (Supplementary Table 6).

### HPV status

HPV status was either determined with p16 immunostaining followed by HPV DNA PCR on p16-positive samples (AC1) and/or with HPV16 E6*I RT-PCR in the AC1 and qPCR cohorts. Both assays have been validated and described before [26]. In AC2, the HPV status was not available. In the other cohorts on the other hand, 1 out of 151 (AC1) and 1 out of 126 (qPCR cohort) tumors were HPV-positive. Hence, the contribution of HPV positive tumors in AC2 was assumed to be low and no samples were excluded.

### Gene expression datasets

Similarly preprocessed VUmc (GSE84846) and UMCU (GSE30788) microarray datasets were combined, and comparability of the expression data of both centers was ensured. Data from AC2 (GSE84846) were not combined to the other datasets, because of a different reference design: Universal Human Reference RNA (cat. 740000, Agilent Technologies, Santa Clara, CA, USA) in AC1 and a pool of cell line RNA in AC2 (CAL 27, ATCC CRL-2095, American Type Culture Collection, Manassas, VA, USA). All preprocessing steps of the microarray data were performed using the limma package [27] in R (Supplementary Materials).

### RT-qPCR

RNA was purified from fresh frozen tumor tissue and synthesis of cDNA was performed from 1 μg of total RNA using the High-Capacity RNA-to-cDNA Kit (cat. 4387406, Applied Biosystems; Foster City, CA). qPCR was performed using Taqman Low-Density Array (TLDA) Cards (cat. 4346800, Applied Biosystems) (Supplementary Table 2). qPCR Ct values were determined with predefined thresholds that were equal per gene for all patients. Relative gene expression was determined by the ΔΔCt method [28] using GUSB Ct-values for normalization. GUSB was selected as the most stable housekeeping gene (see Supplementary Table 7) out of four candidate genes (GAPDH, GUSB, RPLP0, and RPL4).

### Statistical analyses

Per dataset, the predictive power for LNM and survival was assessed with the global test [29, 30]. Datasets with significant predictive power (p <0.05) were used for gene selection. Genes were selected from the microarray data by using a combination (detailed later) of lasso logistic regression or lasso Cox regression and univariable FDR-based association analysis. The latter was included to enhance reproducibility of individual markers assayed by qPCR. The gene selection procedure is displayed in Figure 1 and further detailed in the Supplementary Materials. For the LNM genes, the p-values per gene of AC1 and AC2 were combined by Fisher's combined probability test, whereas for the prognostic genes only p-values of AC1 were considered, because the AC2 data did not pass the global test. For technical validation, the correlation between microarray and RT-qPCR data of 20 cases was determined by Pearson's correlation coefficient. For the RT-qPCR data, the univariable association of delta Ct values of the selected genes with either LNM or OS was determined by logistic or Cox regression, respectively. For prediction on independent samples, clinical variables were selected using stepwise regression, followed by adding the selected genes in a logistic (Cox) ridge regression to render multi-type prediction models. Model performance was assessed by bootstrapping. The prediction models for outcome consisted of (1) prognostic genes, (2) significant clinical factors and pathological TNM-stage (pTNM), (3) significant clinical factors and a composite pathological variable (positive if ECS or R+ surgical margins or >1 LNM was present), and the combinations (4) 1+2 and (5) 1+3. The predictive performance was assessed by area-under-the-ROC-curve (AUC) and integrated AUC (iAUC) over 5-year follow-up time for LNM and OS, respectively, complemented for LNM by the negative predictive value (NPV). Additive value of the gene signature was assessed with the global test. All statistical tests performed were two-sided. Univariable p-values were corrected for multiple testing using the Benjamini-Hochberg FDR procedure [31].

## SUPPLEMENTARY MATERIALS FIGURES AND TABLES













## Abbreviations

AC1: array cohort 1
AC2: array cohort 2
AUC: area-under-the-ROC-curve
cDNA: complementary DNA
CI: confidence interval
DFS: disease-free survival
ECS: extracapsular spread
FDR: false discovery rate
FFPE: formalin-fixed paraffin-embedded
HNSCC: head and neck squamous cell carcinoma
HPV: human papillomavirus
iAUC: integrated AUC
LNM: lymph node metastasis
LNMsig: lymph node metastasis signature
NPV: negative predictive value
OPSCC: oropharyngeal squamous cell carcinoma
OS: overall survival
OSCC: oral squamous cell carcinoma
OSsig: survival signature
pCompVar: pathology composite variable
pTNM: pathological TNM-stage
PY: packyears
qPCR: quantitative real-time polymerase chain reaction
R+: tumor-positive surgical margins
SD: standard deviation
SNB: sentinel node biopsy
TCGA: The Cancer Genome Atlas
UHD: University Hospital Düsseldorf
UHPMC: University Hospital Parma Medical Center
UMCU: University Medical Center Utrecht
VUmc: VU University Medical Center

## References

[1] Ferlay J, Soerjomataram I, Dikshit R, Eser S, Mathers C, Rebelo M, Parkin DM, Forman D, Bray F (2015). Cancer incidence and mortality worldwide: sources, methods and major patterns in GLOBOCAN 2012. Int J Cancer.

[2] Braakhuis BJ, Leemans CR, Visser O (2014). Incidence and survival trends of head and neck squamous cell carcinoma in the Netherlands between 1989 and 2011. Oral Oncol.

[3] Gillison ML, Chaturvedi AK, Anderson WF, Fakhry C (2015). Epidemiology of human papillomavirus–positive head and neck squamous cell carcinoma. J Clin Oncol.

[4] Castellsagué X, Alemany L, Quer M, Halec G, Quirós B, Tous S, Clavero O, Alòs L, Biegner T, Szafarowski T, Alejo M, Holzinger D, Cadena E (2016). HPV involvement in head and neck cancers: comprehensive assessment of biomarkers in 3680 patients. J Natl Cancer Inst.

[5] Braakhuis BJ, Snijders PJ, Keune WJ, Meijer CJ, Ruijter-Schippers HJ, Leemans CR, Brakenhoff RH (2004). Genetic patterns in head and neck cancers that contain or lack transcriptionally active human papillomavirus. J Natl Cancer Inst.

[6] O’Sullivan B, Huang SH, Su J, Garden AS, Sturgis EM, Dahlstrom K, Lee N, Riaz N, Pei X, Koyfman SA, Adelstein D, Burkey BB, Friborg J (2016). Development and validation of a staging system for HPV-related oropharyngeal cancer by the International Collaboration on Oropharyngeal cancer Network for Staging (ICON-S): a multicentre cohort study. Lancet Oncol.

[7] Brierley JD, Gospodarowicz MK, Wittekind C (2016). TNM Classification of Malignant Tumours, 8th Edition.

[8] Chinn SB, Myers JN (2015). Oral cavity carcinoma: current management, controversies, and future directions. J Clin Oncol Off J Am Soc Clin Oncol.

[9] Leemans CR, Braakhuis BJ, Brakenhoff RH (2011). The molecular biology of head and neck cancer. Nat Rev Cancer.

[10] Chung CH, Parker JS, Ely K, Carter J, Yi Y, Murphy BA, Ang KK, El-Naggar AK, Zanation AM, Cmelak AJ, Levy S, Slebos RJ, Yarbrough WG (2006). Gene expression profiles identify epithelial-to-mesenchymal transition and activation of nuclear factor-kappaB signaling as characteristics of a high-risk head and neck squamous cell carcinoma. Cancer Res.

[11] De Cecco L, Bossi P, Locati L, Canevari S, Licitra L (2014). Comprehensive gene expression meta-analysis of head and neck squamous cell carcinoma microarray data defines a robust survival predictor. Ann Oncol.

[12] Jung AC, Job S, Ledrappier S, Macabre C, Abecassis J, de Reyniès A, Wasylyk B (2013). A poor prognosis subtype of HNSCC is consistently observed across methylome, transcriptome, and miRNome analysis. Clin Cancer Res.

[13] Lohavanichbutr P, Méndez E, Holsinger FC, Rue TC, Zhang Y, Houck J, Upton MP, Futran N, Schwartz SM, Wang P, Chen C (2013). A 13-gene signature prognostic of HPV-negative OSCC: discovery and external validation. Clin Cancer Res.

[14] Tan PK, Downey TJ, Spitznagel EL, Xu P, Fu D, Dimitrov DS, Lempicki RA, Raaka BM, Cam MC (2003). Evaluation of gene expression measurements from commercial microarray platforms. Nucleic Acids Res.

[15] Roepman P, Wessels LF, Kettelarij N, Kemmeren P, Miles AJ, Lijnzaad P, Tilanus MG, Koole R, Hordijk GJ, van der Vliet PC, Reinders MJ, Slootweg PJ, Holstege FC (2005). An expression profile for diagnosis of lymph node metastases from primary head and neck squamous cell carcinomas. Nat Genet.

[16] Roepman P, Kemmeren P, Wessels LF, Slootweg PJ, Holstege FC (2006). Multiple robust signatures for detecting lymph node metastasis in head and neck cancer. Cancer Res.

[17] van Hooff SR, Leusink FK, Roepman P, Baatenburg de Jong RJ, Speel EJ, van den Brekel MW, van Velthuysen ML, van Diest PJ, van Es RJ, Merkx MA, Kummer JA, Leemans CR, Schuuring E (2012). Validation of a gene expression signature for assessment of lymph node metastasis in oral squamous cell carcinoma. J Clin Oncol.

[18] Schilling C, Stoeckli SJ, Haerle SK, Broglie MA, Huber GF, Sorensen JA, Bakholdt V, Krogdahl A, von Buchwald C, Bilde A, Sebbesen LR, Odell E, Gurney B (1990). Sentinel European Node Trial (SENT): 3-year results of sentinel node biopsy in oral cancer. Eur J Cancer Oxf Engl.

[19] Cancer Genome Atlas Network (2015). Comprehensive genomic characterization of head and neck squamous cell carcinomas. Nature.

[20] Den Toom IJ, Heuveling DA, Flach GB, van Weert S, Karagozoglu KH, van Schie A, Bloemena E, Leemans CR, de Bree R (2015). Sentinel node biopsy for early-stage oral cavity cancer: the VU University Medical Center experience. Head Neck.

[21] Alkureishi LW, Ross GL, Shoaib T, Soutar DS, Robertson AG, Thompson R, Hunter KD, Sorensen JA, Thomsen J, Krogdahl A, Alvarez J, Barbier L, Santamaria J (2010). Sentinel node biopsy in head and neck squamous cell cancer: 5-year follow-up of a European multicenter trial. Ann Surg Oncol.

[22] Civantos FJ, Zitsch RP, Schuller DE, Agrawal A, Smith RB, Nason R, Petruzelli G, Gourin CG, Wong RJ, Ferris RL, El Naggar A, Ridge JA, Paniello RC (2010). Sentinel lymph node biopsy accurately stages the regional lymph nodes for T1-T2 oral squamous cell carcinomas: results of a prospective multi-institutional trial. J Clin Oncol.

[23] Leusink FK, van Es RJ, de Bree R, Baatenburg de Jong RJ, van Hooff SR, Holstege FC, Slootweg PJ, Brakenhoff RH, Takes RP (2012). Novel diagnostic modalities for assessment of the clinically node-negative neck in oral squamous-cell carcinoma. Lancet Oncol.

[24] Pramana J, Pimentel N, Hofland I, Wessels LF, van Velthuysen ML, Atsma D, Rasch CR, van den Brekel MW, Begg AC (2007). Heterogeneity of gene expression profiles in head and neck cancer. Head Neck.

[25] McShane LM, Altman DG, Sauerbrei W, Taube SE, Gion M, Clark GM (2005). Reporting recommendations for tumor marker prognostic studies. J Clin Oncol.

[26] Smeets SJ, Hesselink AT, Speel EJ, Haesevoets A, Snijders PJ, Pawlita M, Meijer CJ, Braakhuis BJ, Leemans CR, Brakenhoff RH (2007). A novel algorithm for reliable detection of human papillomavirus in paraffin embedded head and neck cancer specimen. Int J Cancer.

[27] Ritchie ME, Phipson B, Wu D, Hu Y, Law CW, Shi W, Smyth GK (2015). limma powers differential expression analyses for RNA-sequencing and microarray studies. Nucleic Acids Res.

[28] Livak KJ, Schmittgen TD (2001). Analysis of relative gene expression data using real-time quantitative PCR and the 2(-Delta Delta C(T)) Method. Methods San Diego Calif.

[29] Goeman JJ, van de Geer SA, de Kort F, van Houwelingen HC (2004). A global test for groups of genes: testing association with a clinical outcome. Bioinformatics.

[30] Goeman JJ, Oosting J, Cleton-Jansen AM, Anninga JK, van Houwelingen HC (2005). Testing association of a pathway with survival using gene expression data. Bioinformatics.

[31] Benjamini Y, Hochberg Y (1995). Controlling the false discovery rate: a practical and powerful approach to multiple testing. J R Stat Soc Ser B Methodol.

