# Supplementary material for: Prognostic modeling of oral cancer by gene profiles and clinicopathological co-variables
Source: Oncotarget. 2017 Jul 26;8(35):59312–23. doi: 10.18632/oncotarget.19576 (PMC5601734; doi:10.18632/oncotarget.19576)
Supplement: Supplementary file 1 [file oncotarget-08-59312-s001.pdf]

Prognostic modeling of oral cancer by gene profiles and clinicopathological co-variables

SUPPLEMENTARY MATERIALS

Patients

Four independent cohorts of oral squamous cell carcinoma (OSCC) patients with frozen biopsies were included in this study: (1) a cohort of 150 patients from the University Medical Center Utrecht (UMCU) and VU University Medical Center Amsterdam (VUMC) for microarray gene expression profiling (array cohort 1, AC1); (2) a cohort of 99 patients from the University Hospital Parma Medical Center (UHPMC) for microarray gene expression profiling (array cohort 2, AC2); (3) a cohort of 125 patients from VUMC, UHPMC and University Hospital Düsseldorf (UHD) for qPCR gene expression profiling; and (4) an RNAseq dataset of HPV-negative OSCC tumors from the Cancer Genome Atlas Network publication [1]. Inclusion criteria were: presentation with a squamous cell carcinoma in the oral cavity, date of incidence prior to July 1st 2012, and treated surgically with curative intent. All patients were 18 years or older and had no previous malignancy that impacts outcome. Included ICD-10 codes were: C00.3-4, C02.0-3, C02.8-9, C03, C04, C05.0, and C06. Human papilloma virus (HPV) positive tumors were excluded from further analysis. Informed consent was obtained of enrolled patients when required, and nation- and institution-specific procedures and guidelines were followed in addition. For instance, for the Netherlands use of residual tissue from surgical specimen adhered to the Code of conduct for responsible use by the Federation of Dutch Medical Scientific Societies (FDMSS). This study followed the Guidelines for the REporting of tumor MARKer Studies (REMARK) [2](Supplementary Table 6).

Specimen

Biopsies were taken from the surgical specimen at time of surgery, snap frozen and subsequently stored in liquid nitrogen. Five to ten 20 µm sections were used for RNA isolation. Before and after sampling of the 20 µm sections, 5 µm sections were made and stained by haematoxylin and eosin to ensure that at least 50% tumor cells were present in the biopsy. RNA isolation was performed using TRIzol (cat. 15596026, Life Technologies, Breda, The Netherlands; AC1) or with columns using the RNeasy Mini Kit (cat. 74104, Qiagen, Hilden, Germany; AC2 and qPCR cohort), according to the protocol of the suppliers. HPV status was either determined with p16 immunostaining followed by HPV DNA PCR on p16-positive samples (AC1) or with HPV16 E6*I RT-PCR (qPCR cohort) in the AC1 and AC3 cohorts. Both assays have been validated and described before [3]. In AC2, the HPV status was not determined. In the other cohorts on the other hand, 1 out of 151 (AC1) and 1 out of 126 (qPCR cohort) tumors were HPV-positive. Hence, the contribution of HPV positive tumors in AC2 was assumed low and no further samples were excluded. Quantity and quality of the RNA was tested with the Nanodrop (cat. ND-1000, Thermo Fisher Scientific, Amsterdam, The Netherlands) and the Bioanalyzer 2100 (cat. G2939AA, Agilent Technologies, Amstelveen, The Netherlands) using the RNA Nanokit (cat. 5067-1511, Agilent). RNA Integrity Numbers (RIN-value) were between 6.3 and 10.0.

Expression arrays

Two independent cohorts of OSCC patients were processed for gene expression microarray analysis: (1) a cohort of 2 merged tumor gene expression profiles (array cohort 1, AC1); and (2) array cohort 2 from the University Hospital Parma Medical Center (UHPMC). In AC1, array hybridization was performed, using 0.5 µg total RNA in the Low RNA Input Linear Amplification Kit (cat. 5184-3523, Agilent) and the 4x44K Whole Human Genome Arrays, according to the manufacturer (Agilent) using dual color labeling. The handling of the UMCU samples and their RNA isolation has been published before [4]. Microarray data of UMCU were retrieved from the Gene Expression Omnibus (GSE30788). Additional information on these samples was obtained from Agendia (Amsterdam, the Netherlands). In AC2, 0.2 µg of total RNA was labeled and simultaneously amplified following the "Two-Color Microarray-Based Gene Expression Analysis (Quick Amp Labeling) Protocol" (Agilent Technologies). Labeled-amplified RNA samples were then hybridized on 4X44K Whole Human Genome DNA microarray slides (cat. G4112F, Agilent) according to the instructions of the manufacturer. An Agilent Technologies Scanner G2505B US45102976 and the Feature Extraction (FE) Software v 9.5.1.1 with the GE2- v5_95_Feb07 Gene Expression protocol were used to scan microarray slides and extract data, respectively.

Preprocessing microarray data

AC1 gene expression data generated at VUmc was preprocessed in an identical way as those previously preprocessed at UMCU [4]. This comprised: 1) extraction of the median signal from the raw data files without background correction, and 2) median and loess within-array normalization as implemented in the Limma-package (http://www.bioconductor.org). Preprocessed VUmc and UMCU data sets were combined by limiting both to the probes that overlapped (using their Agilent probe identifiers). Finally, comparability of the expression data of both centers was ensured by a) joint between-array normalization (Aquantile as implemented in the Limma-package), and b) removal of possible batch (i.e. center) effects using the Combat-package [5]. Raw and processed data are publicly available in the gene expression omnibus (GEO) database (GSE85446).

Data from AC2 were not combined to the other datasets, because of a different reference design: Universal Human Reference RNA (cat. 740000, Agilent Technologies, Santa Clara, CA) in AC1 and a pool of cell line RNA in AC2 (CAL 27, ATCC CRL-2095, American Type Culture Collection, Manassas, VA). AC2 data were preprocessed in the same way as data set AC1. Probes with more than 20% missing values were deleted. AC2 consisted of 106 samples, but seven were excluded because of poor quality MA plots. Remaining missing values were imputed with nearest neighbor averaging with R package impute. For the LNM analysis, two additional patients were excluded because information on the LNM was missing. Raw and processed data are registered in the gene expression omnibus (GEO) database (GSE84846), but are not publicly available until July25th, 2018 or after publication of this manuscript.

Gene selection

Per data set, the predictive significance for LNM and survival was assessed with the global test [6, 7]. Data sets with significant results ($p < 0.05$) were used for gene selection. For prediction of LNM, we obtained significant results for both AC1 and AC2 (AC1, p=9.3E-06; AC2, p=9.9E-03), hence both were used for gene selection. For prediction of survival, only AC1 showed predictive significance (AC1, p=7.8E-3; AC2, p=0.73) and was used for further analysis. This difference is likely explained by a shorter follow-up time in AC2 compared to AC1 (AC1, mean overall follow-up time: AC1: 4.7 years (SD=3.2), AC2: 3.0 years (SD=1.5)).

The selection of genes was based on univariable and multivariable analysis with an equal contribution to the final signature (50% of genes from univariable analysis, 50% from multivariable analysis). For the multivariable selection our aim was to come to an optimal set of genes that orthogonally contribute on top of each other (see Supplementary Example R code below). Due to selection from a large number of genes using limited sample series, it is to be expected that some genes will not validate. Also, the platform transition we conducted increases the probability that genes can not be validated, although this is somewhat counterbalanced by the technical validation. For this reason we planned some redundancy in the selected genes. This is best achieved by selecting those genes that have the highest signal to noise ratio (e.g. low p-value). The total number of selected genes was limited to 60 genes of interest because of the chosen qPCR array card design (60 target genes + 4 housekeeping genes, 3 replicates, and 2 samples / array card). Univariable analysis included t-tests (LNM) and Cox regression (overall survival). For LNM p-values of AC1 and AC2 were combined with Fisher's combined p-value. P-values were adjusted with the Benjamini-Hochberg procedure to control the false discovery rates (FDR) [8]. Multivariable analysis consisted of lasso logistic regression (LNM) and lasso Cox regression (overall survival), as implemented in package glmnet in R. To stabilize the selection, the lasso was run repeated N times per analysis (as a leave-one-out cross-validation (LOOCV)). For LNM, the selection frequencies of AC1 and AC2 were added up. We next selected those genes that were used most often in the models. For survival, models were tested with and without addition of pathological stage of disease (pTNM) and age as unpenalized covariate, but the additions did not change the list of selected genes considerably.

To reduce dimensionality and enrich for relevant predicting genes, previously published HNSCC gene signatures were used as input for gene selection. For LNM, we used a previously published LNM predicting gene profile [9, 10] consisting of 732 probes, which was later validated in a multicenter trial [4]. For survival, thoroughly validated prognostic gene signatures were missing. Therefore, we combined a set of 9 prognostic gene expression profiles [11-19] (1,426 probes) and an in-house discovered prognostic profile of genes for which copy number alterations and gene expression were best correlated (348 probes). This combined survival profile consisted of 1,762 probes. Twenty genes were selected from the combined survival profile (1,762 probes), and 20 genes were selected from analyses that included all probes (37,622 probes in AC1).

Quantitative real-time PCR

A 384-well Taqman Low-Density Array (TLDA) Card was designed with the selected 60 prognostic genes + 4 housekeeping genes (GAPDH, GUSB, RPL4, RPLP0). Each gene expression assay was represented by 3 replicates. The initial design (TLDA.v1) and optimized design (TLDA.v2) after technical validation (see below)

are shown in Supplementary Table 2. One µg of mRNA was treated with DNAse I, Amplification Grade (cat. 18068015, Invitrogen; Carlsbad, CA) in a 10 µl reaction volume. The DNAse-treated mRNA was subsequently used for cDNA synthesis with the High-Capacity RNA-to-cDNA Kit (cat. 4387406, Applied Biosystems; Foster City, CA) in a 24.4 µl reaction volume. The qPCR reaction mix consisted of (1) 20 µl cDNA (819 µg), (2) 190 µl water, and (3) 210 µl 2X TaqMan Gene Expression Master Mix (cat. 4369016, Applied Biosystems). Subsequently, the reaction mix was loaded on the TLDA cards according to the protocol of the supplier. Reaction mixes of 2 samples were loaded per TLDA card. Experiments were performed on an ABI Prism 7900HT Fast Real-Time PCR System (cat. 4329001, Applied Biosystems). Thermal cycling conditions were: 50°C for 2 minutes; 94.5°C for 10 minutes; 40 cycles of denaturation at 97°C for 30 seconds and annealing and extension at 59.7°C for 1 minute. The median result of a triplicate assay was used in downstream analysis.

Gene expression analysis from qPCR data

For each qPCR reaction, the Ct-value was determined as the cycle number at which the fluorescence signal reached a fixed threshold using the SDS RQ Manager Version 1.2.2 (Applied Biosystems). Next, the Ct-values were normalized to the GUSB expression level, which was the most stable housekeeping gene in this dataset. To select the most stable housekeeping gene, we determined the standard deviation of the gene expression within all samples and correlation of the housekeeping gene to the average gene expression of all target genes (Supplementary Table 7). GUSB had both the lowest SD (SD=0.94) and the highest correlation to the average expression of all target genes (r=0.77).

Technical qPCR validation

The 60 selected target genes were technically validated using a subset of 20 cases from AC2 to evaluate the platform transition. Hence, the qPCR data were correlated to the array data of the same samples. These 20 cases were selected to contain a 1:1 ratio of N0/N+ cases and patients that survived/deceased. For these 20 cases, Pearson's correlation coefficients were calculated between microarray and corresponding qPCR data (Supplementary Table 3). Poor correlation was defined as an r of >1 SD below the mean and a p-value >0.1 (because of the small sample size). Logistic and Cox regression analyses were performed to determine the predictive performance of the genes in this cohort.

Histopathology

Formalin-fixed paraffin embedded slides of the surgical specimens were examined by two specialized pathologists (EB + EMS), according to the guidelines of the Royal College of Pathologists UK (https://www.rcpath.org/resourceLibrary/dataset-for-histopathology-reporting-of-mucosal-malignancies-of-the-oral-cavity.html). Tumors were staged according to TNM classification of Malignant Tumors, 7th Edition, published in affiliation of the Union for International Cancer Control (UICC). The margin status was evaluated and divided into three groups: (1) involved margins when carcinoma was present in or within 1 mm of the margin, (2) negative margins when the excised carcinoma was > 5 mm from the surgical margin, and (3) close margins when the tumor was 1-5 mm from the surgical margin [20]. For model building, the margin status was subsequently subdivided in two groups: involved margins when carcinoma was present in or within 1 mm of the margin (R+); or negative margins, when the excised carcinoma was > 1 mm from the surgical margin (R0). The presence of lymph node metastasis (LNM) was determined by standard histopathological examination of the neck dissection specimen if present. When the neck was left untreated, two scenarios were possible: a patient was diagnosed N+ when a delayed lymph node metastasis developed during follow-up (≤ three years after treatment) or remained N0 when not. In the different cohorts, 128 of 150 (85.3%, AC1), 89 of 99 (89.9%, AC2) and 103 of 125 (82.4%, qPCR cohort) the neck was treated with a primary neck dissection. Extracapsular spread (ECS) was present if the tumor extended beyond the capsule of the lymph node. When there was doubt, the case was classified as having ECS according to the guidelines of the Royal College of Pathologists UK [21]. We created a pathological composite variable (pCompVar) that was scored positive if ECS or R+ or >1 LNM was present.

Clinical data

Several clinical variables were used for prognostic model building. These included age at diagnosis, gender, smoking behavior in packyears (1 packyear equals 20 cigarettes a day during 1 year), ECOG Performance Status [22], and comorbidity. Comorbidity was classified using the Adult Comorbidity Evaluation 27 (ACE-27) [23], in which an overall comorbid score is graded in four levels: none, mild, moderate or severe. For smoking we only considered the packyears in the model building. Compared to categorical smoking variables, packyears contains the most information about the smoking habits and was also the most significant smoking variable for OS.

Outcomes

Overall survival (OS) was defined as time from date of incidence to death from any cause. Disease free survival (DFS) was defined as time from date of incidence to development of locoregional recurrence, distant metastasis or second primary HNSCC. Mean survival times for the various data sets were calculated with the reverse Kaplan-Meier as suggested by Schemper et al. [24] Patients who died of other causes or develop second primary tumors outside the head and neck region (SPT), were censored on the date of death or incidence date of the SPT. Local recurrences were scored when these developed within two centimeters of the index tumor and within three years after therapy, whereas a regional recurrence was documented when it developed in a treated neck within three years after treatment.

Statistical analysis of the RT-qPCR dataset

For the qPCR data, the univariable association of delta Ct values of the selected genes with either LNM or OS/DFS was determined with logistic or Cox regression, respectively. Multivariable models with the selected genes were made with logistic ridge regression (LNM) or Cox ridge regression (OS/DFS). For the clinical variables univariable p-values of clinical variables were determined by Cox proportional hazards regression (OS, DFS) or logistic regression (LNM). Patients with moderate and severe comorbidity (ACE-27) were considered as one group in the analysis, because the group with severe comorbidity (ACE-27) was very small. TNM-stage was dichotomized as early stage disease (pTNM I+II) and advanced stage disease (pTNM III+IV). Variables with p-value lower than 0.15 were considered as candidates for a multivariable model. For LNM prediction, no clinical variables met this criterion. Next, stepwise regression was performed to identify a multivariable model with clinical variables (using procedure 'step' in R). Stepwise selection with Akaike Information Criterion (AIC) was performed and in each step a variable was added or dropped, which identified the best model. For OS, the stepwise selection procedure selects age and packyears. The prediction models for outcome consisted of (1) prognostic genes only, (2) clinical variables and pathological TNM-stage (pTNM), (3) clinical variables and a composite pathological variable (positive if ECS or R+ or >1 LNM was present), and the combinations (4) 1+2 and (5) 1+3. In combined clinical and genomic models, the clinical variables were not penalized and the genes were incorporated with a ridge penalty to avoid overfitting. The predictive performance was measured by area-under-the-ROC-curve (AUC) and integrated AUC (iAUC) [25] at 5-year follow-up time for LNM and OS/DFS, respectively, complemented for LNM by the NPV, i.e. the proportion of true negatives among all negative tests. Model performance was assessed by bootstrapping, confidence intervals around the AUC, sensitivity, specificity, PPV, and NPV were calculated according to methods described by Jiang et al. [26-28] (see Supplementary Outline of Statistics and Supplementary Example R code). Model performance only takes into account the uncertainty in the genomic, clinical, and pathological coefficients. The variable selection of clinical variables was not bootstrapped, the pathological variables (pTNM and CompVar) were selected based on their known clinical relevance. For the genomics variables we did not perform further selection on the RT-qPCR data. For OS and RFS the subgroup analysis was performed by refitting the model to the cases of that subgroup. For LNM subgroup analysis, refitting was not possible due to the small sample size of the subgroup (n=54) with a low number of cases (15). Therefore, the AUC for the subgroup was computed by first fitting the model to all cases (n=125), and then considering the subgroup. Additive value of the gene signature was assessed with the global test [6, 7]. All statistical tests performed were two-sided. In multiple testing settings, univariable p-values were corrected using the Benjamini-Hochberg FDR procedure [8].

External validation with TCGA RNAseq dataset

Only the 279 patients that were included in the Cancer Genome Atlas Network publication [1] were used for this analysis, because the RNAseq derived HPV-status, which was considered the most accurate, was not available for the other cases. Of these cases, the normalised RNASeqv2 TCGA data were downloaded with R package TCGA2STAT. Additional clinical information was downloaded directly from the Broad Institute (http://gdac.broadinstitute.org/runs/awg_hnsc__2013_03_30/reports/cancer/HNSC-TP-HPV-negative-36/correlate/pick_tier1_cdes/nozzle.html). Only HPV-negative tumors of the oral cavity were considered (n=160). For lymph node metastasis, patients with a pathological NX-stage were excluded. This left 133 patients for lymph node metastases (LNM). For OS, the survival time of one patient was missing and 159 patients were available for analysis. The LNM outcome was defined as having a pathological N-stage larger than 0. Of the 60 genes selected on the microarray data, 1 gene could not be matched to the TCGA data (LRCOL1).

Before analysis the data were transformed by taking the square root and scaling the data (e.g. all genes transformed to zero mean and unit variance). For OS and for LNM, the global test was used to assess the association with the genomic signatures. Further predictive performance was assessed by fitting and bootstrapping a logistic (for LNM) and Cox (for OS) regression model with ridge penalization.

Sample size considerations

Exact sample size calculations are inherently difficult for ridge regression. They also require knowledge of the effect sizes, which are unknown in our multivariate setting.

To provide some insights regarding correct sample size, we performed some ad hoc tests with the qPCR cohort. Random samples of 45-115 patients were repeatedly drawn (50 times) without replacement from the data, supplemented with the actual data for n=125. For each outcome variable, the global test was used to assess the predictive performance of the genomic signatures. For each same sample size we took the medium p-value across the random samples. Sample sizes of 85 cases and more gave consistent p-values <0.05 for each outcome variable (see Supplementary Figure 6), assuring that the sample size of 125 is sufficient to assess the performance of the signatures.

Relation of the genomic signatures to other prognostic markers

We additionally analyzed the association between the prognostic markers and the OSsig (here taken as the linear predictor of the Cox ridge regression, i.e. the log of the hazard ratios), as recommended in REMARK criterion 14 (Supplementary Table 6). In this analysis we considered the variables used in the prognostic models (i.e. age, smoking, pTNM, pCompVar) and additionally considered gender and comorbidity (ACE27). For the age and packyears, a Pearson's correlation coefficient and an additional p-value were calculated. For sex, pTNM, and pCompVar we performed a t-test and for ACE27 we performed an ANOVA. Significant correlation between OSsig and gender, smoking, ACE27 and pCompVar were found (Supplementary Figure 7). However, as can be seen in this figure, the size of the effects was small. Secondly, we assessed the iAUC of the genomic predictor in different subgroups (Supplementary Table 8). Subgroups included were: (1) pCompVar negative and positive; (2) Age <70 and ≥70 years at diagnosis; (3) Smoking: packyears <median and ≥median value in this dataset; (4) Comorbidity (ACE27): ACE27 0-1 and ACE27 2-3; (5) Male and female gender; and (6) pathological stage I+II and III+IV. This analysis showed that the OSsig has good discriminative power in the various subgroups.

SUPPLEMENTARY OUTLINE OF STATISTICS

1. Gene selection with microarray data.
    a. Univariable selection
        i. p-value per gene with false discovery rate (FDR) control.
            1. Welch t-test for LNM
            2. Cox regression for OS
    b. Multivariable selection with lasso (see example code)
        i. Repeatedly fitting the lasso. Genes are ranked based on their selection frequency across the lasso fits (see example code of the lasso selection).
        ii. Analysis conducted with and without mandatory covariates. Mandatory covariates were not subject to the lasso penalty (e.g. unpenalized).
        iii. Logistic regression for LNM, Cox regression for OS
2. Technical validation
    a. Pearson's correlation coefficient (and associated p-value) between qPCR – microarray data per gene.
3. Validate selected genes on independent data (i.e. qPCR data).
    a. Univariable assessment
        i. p-value per gene with false discovery correction (FDR) control.
            1. Logistic regression for LNM
            2. Cox regression for OS/RFS
4. Fit with selected genes on the (independent) qPCR data.
    a. Low dimensional models (i.e. clinical and/or pathological) fitted with standard regression techniques.
        i. Clinical variable are selected on the qPCR data for OS. First we conducted a univariate screening with p-value of 0.15. Next the clinical model was made by stepwise regression based on AIC.
    b. Genomics models fitted with ridge regression
    c. Combined models (e.g. clinical, and/or pathological and/or genomics) for RFS/OS fitted with ridge regression. Clinical and/or pathological are not subject to the ridge penalty.
5. Predictive accuracy of the models is assessed by bootstrapping.
    a. Bootstrap assessed the parameter uncertainty.
    b. For the clinical data the variable selection process was not bootstrapped.
    c. For genomics and pathological variables there was no further variable selection.
    d. For bootstrap code see http://github.com/DennisBeest/BootPredError.
6. Step 4b and step 5 are repeated with the (independent) TCGA data.

SUPPLEMENTARY EXAMPLE R CODE OF THE MULTIVARIABLE GENE SELECTION (LASSO)

```
#######################################################

    #----Example code
    library(glmnet)
    #######################################################
    #----Simulated some survival data, replace these by real data
    #######################################################
    P <- 200
    N <- 100
    Train <- matrix(nrow=N,ncol=P)
    Train[] <- runif(P*N)
    Survival <- rep(c(0,1),each=N/2)
    Train[Survival==1,1:5] <- Train[Survival==1,5]+0.5
    Time <- runif(N)
    Time[Survival==1] <- Time[Survival==1]/2
    colnames(Train) <- 1:P
    penfac <- rep(1,times=P)
    #######################################################
    #----Selection with lasso in the form of a LOOCV.
    #----Alternatively the lasso can be run repeatedly on the whole data, or on bootstraps.
    #----The lasso is likely to select a different set of variables each time it is run on slightly different data or when the cross-validation folds are changed. Especially when the the data are stongly correlated. The aim of running it repeatedly is to stabilise the selection.
    #----An unpenalized covariate can be incorporated by setting penfac to 0 for that variable.
    #######################################################
    N <- length(Survival)        #Number of patients
    Selected <- list()    #List for saving selected genes
    SavePrediction <- numeric()    #Optionally for saving cross-validated predictions
    for(i in 1:N)            #Repeat for each patient
    {
      #######################################################
      #Leave one patient out
      Y <- cbind(time=Time[-i],status=Survival[-i])
      X <- Train[-i,]
      #######################################################
      #Fit lasso
      model <- cv.glmnet(X,Y,family="cox",standardize = FALSE,alpha = 1,nfolds=5,penalty.factor=penfac)
      #######################################################
      #Keep track of selected variables
      betas <- model$glmnet.fit$beta[,model$glmnet.fit$lambda==model$lambda.min]
      Selected[[i]] <- names(betas) [which(betas!=0)]
      #######################################################
      #Optionally get an indication of the predictive value
      XTest <- Train[i,,drop=FALSE]
      SavePrediction[i] <- predict(model,newx=XTest,s=c("lambda.min"))
    }
    #######################################################
    #----Add selected variables together and sort/rank
    tab <- table(unlist(Selected))
    o <- order(tab,decreasing=TRUE)
```

```
print(cbind(tab[o]))
############################################################
#----End of code
```

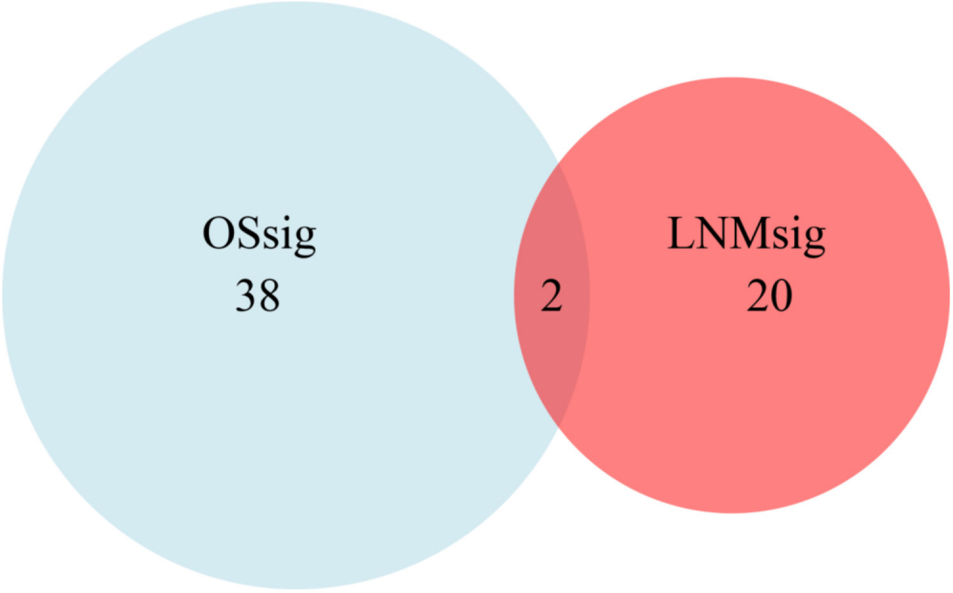

Supplementary Figure 1: Venn diagram of selected gene signatures shows 2 overlapping genes between OSsig and LNMsig. Venn diagram of selected overall survival gene signature (OSsig, 40 genes) and lymph node metastasis gene signature (LNMsig, 22 genes) shows that 2 genes overlap, limiting the combined signatures to 60 genes. LNMsig, lymph node metastasis signature; OSsig, overall survival signature.

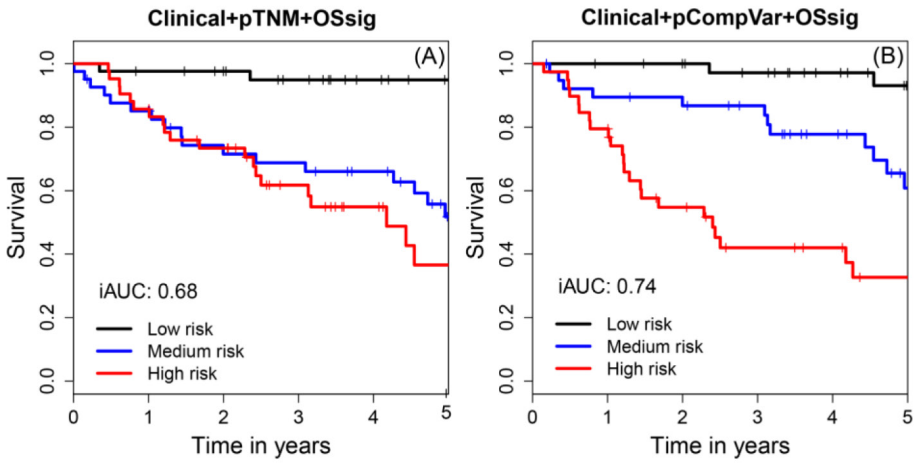

Supplementary Figure 2: Best predicting integrated models of clinical variables, histopathological variables and the overall survival signature (OSsig). (A-B) Results of the Kaplan-Meier analysis are depicted for overall survival in the independent validation cohort using the best predicting models with risk groups defined by tertile predicted hazards of (A) the OSsig combined with significant clinical variables (i.e. age at diagnosis, smoking) and pathological TNM stage (pTNM) (iAUC=0.68, OSsig: P=0.03 (global test)), and (B) the OSsig combined with the same clinical variables and the a composite histopathology variable (pCompVar) that was scored positive if extracapsular spread (ECS) or involved resection margins (R+) or >1 lymph node metastasis was present; all three variables are currently used as indicators for adjuvant treatment (iAUC=0.74, OSsig: P=0.02 (global test)). Area under the curve was integrated over 5 year follow-up time. Tick marks on curves indicate censoring. iAUC, integrated Area Under the Curve; OSsig, overall survival signature; pCompVar, pathological composite variable; pTNM, pathological TNM stage.

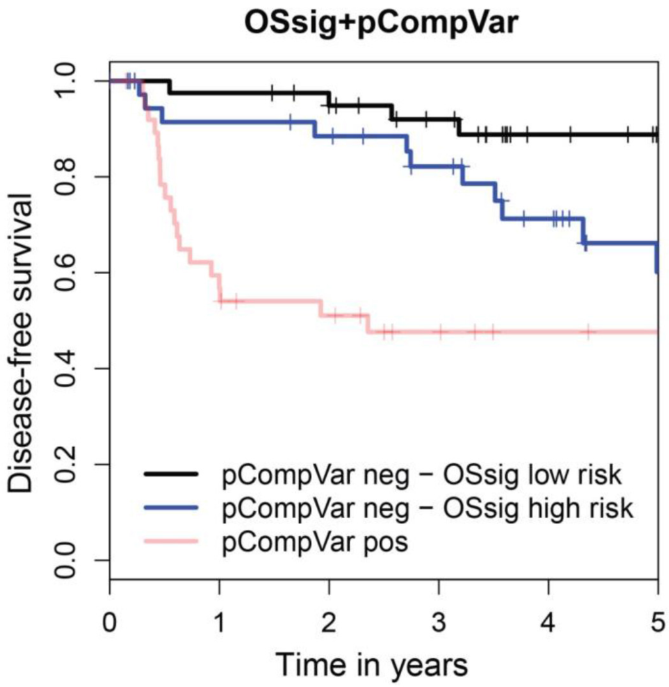

Supplementary Figure 3: The Overall Survival genomic signature (OSsig) predicted disease-free survival of oral squamous cell carcinoma (OSCC) in a subgroup of prognostically favorable patients. A subgroup of 79 prognostically favorable patients was identified based on histopathological variables, i.e. tumor-free margins (R0), ≤ 1 lymph node metastasis (LNM), and without extracapsular spread (ECS-neg). Depicted is a Kaplan-Meier analysis for disease-free survival in these pCompVar-*negative* patients of the independent validation group with risk groups defined by median predicted hazards of the OSsig (black and blue lines; integrated area under the curve (iAUC)=0.65, OSsig: P=7E-3 (global test)). Area under the curve was integrated over 5 year follow-up time. Abbreviations: iAUC, integrated Area Under the Curve; OSsig, Overall Survival signature; pCompVar, pathological composite variable.

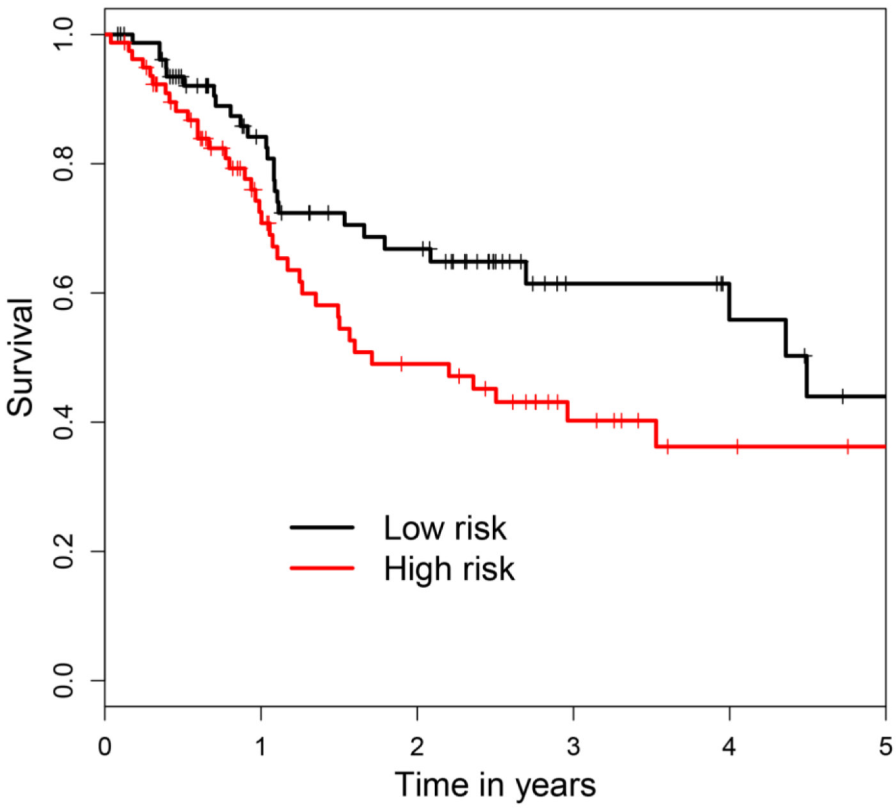

Supplementary Figure 4: Additional external validation of the overall survival signature (OSsig). Kaplan-Meier analysis of overall survival with risk groups defined by median predicted hazards by the OSsig. RNAseq data of the TCGA cohort head and neck squamous cell carcinoma cohort were used of HPV-negative, OSCC patients (n=159; iAUC=0.59; OSsig: P=0.02 (global test)). Area under the curve was integrated over 5 year follow-up time. Tick marks on curves indicate censoring. Abbreviations: iAUC, integrated Area Under the Curve; OSsig, Overall Survival signature.

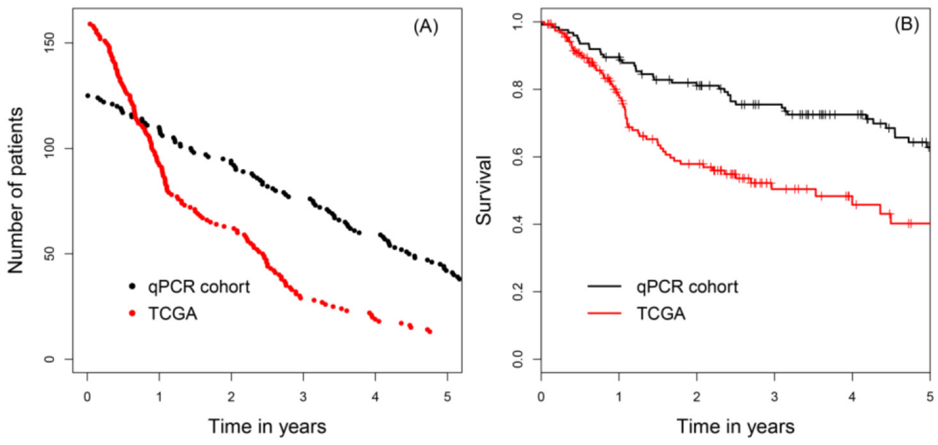

Supplementary Figure 5: Comparison of follow-up time and baseline survival curves shows significant differences between our qPCR validation cohort and the TCGA cohort. (A) Number of patients under consideration (y-axis) in relation to follow-up time (x-axis) of qPCR cohort (black) and TCGA cohort (red). **(B)** Baseline Kaplan-Meier analysis of qPCR cohort (black) and TCGA cohort (red) differed significantly (cox regression: HR= 2.0, 95% CI =1.4–2.9), P=3E-4).

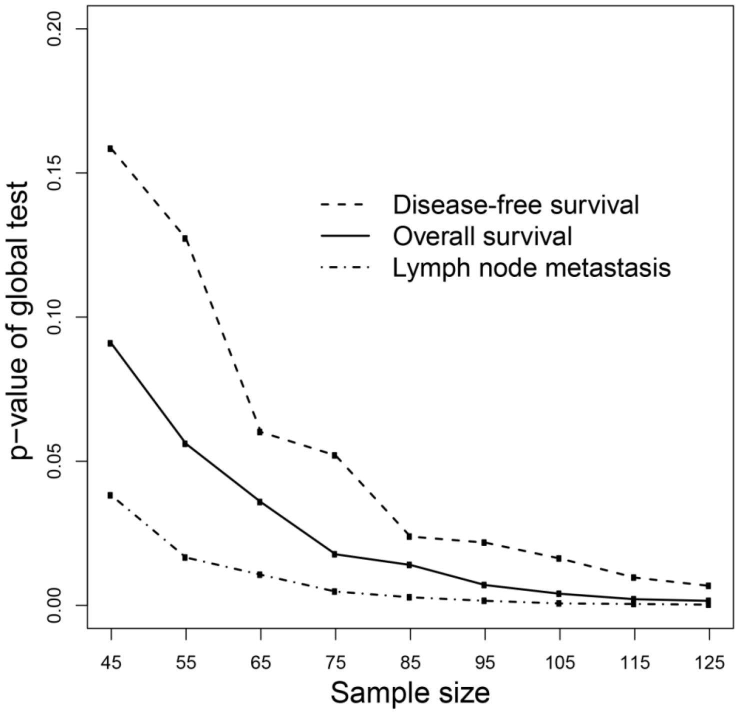

Supplementary Figure 6: Random sampling from independent qPCR cohort shows clear relationship between sample size and global test p-value, and provides rationale for the tested sample size. Random samples of increasing sample size were repeatedly (50 times) drawn without replacement from the data (y-axis). For each outcome variable, the global test was used to assess the predictive performance of the genomic signatures. P-values were averaged between random samples of the same size (x-axis). Sample sizes of 85 cases and more gave consistent p-values <0.05 for each outcome variable, assuring that the sample size of 125 cases of the independent validation cohort should be sufficient to assess the performance of the signatures.

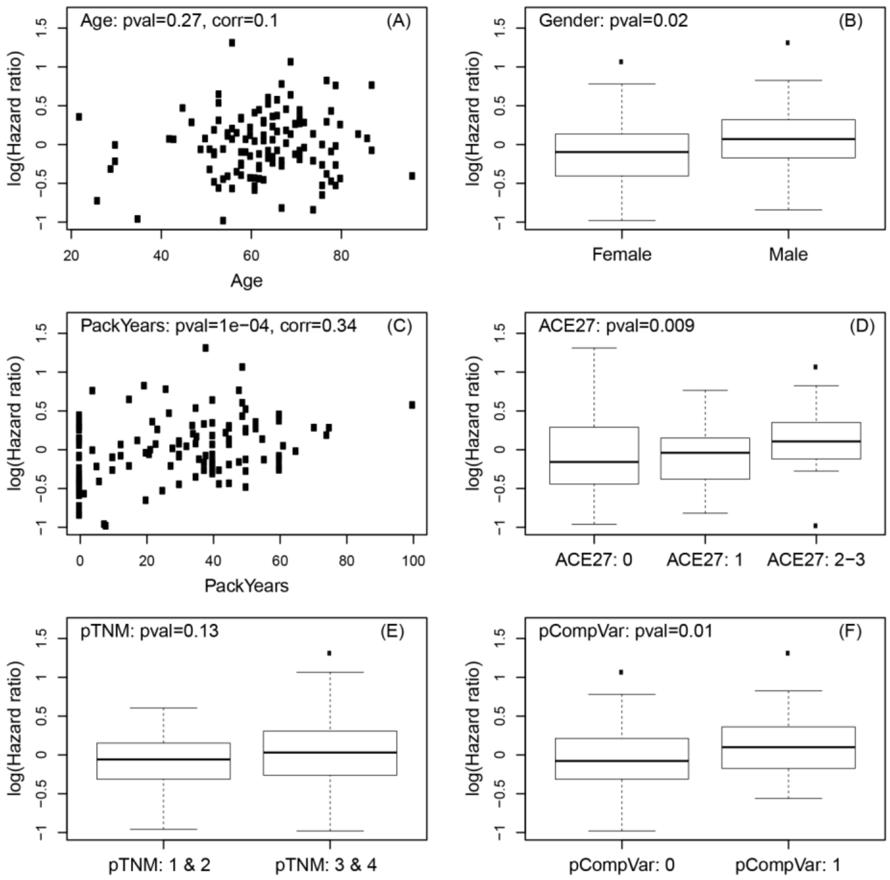

Supplementary Figure 7: Relation of Overall Survival signature (OSsig) to other prognostic markers. (A-F) Relation of the OSsig linear predictor of the Cox ridge regression, i.e. the log of the hazard ratios (y-axis), and other prognostic variables (x-axis). A Pearson's correlation coefficient and an additional p-value were calculated for numerical variables, for ACE27 we performed an ANOVAa, and for the remaining categorical variables, a t-test was performed. Relation between OSsig and (A) age (in years, r=0.1, P=0.27), (B) gender (P=0.02), (C) smoking (in packyears; r=0.34, P=0.0001), (D) comorbidity (ACE27; P=0.009), (E) pathological TNM stage (pTNM; P=0.13), and (F) a composite pathology variable (pCompVar) that was positive if extracapsular spread or tumor-positive resection margins or >1 lymph node metastasis was present (P=0.01). For each marker the size of the effects was small, even when a significant relation was found. corr, Pearson's correlation coefficient; pCompVar, pathological composite variable; pTNM, pathological TNM stage.

Supplementary Table 1: Genes with false discovery rate <0.1 on microarray data

See Supplementary File 1

Supplementary Table 2: Selected gene signature and qPCR assays

See Supplementary File 2

Supplementary Table 3: Pearson's correlation coefficients and corresponding p-values of technical validation

See Supplementary File 3

Supplementary Table 4: Coefficient estimates and false discovery rates of qPCR results of the gene signatures

Supplementary Table 5: Coefficients and p-values of clinical and pathological variables for integration with gene signatures

Supplementary Table 5: Coefficients and p-values of clinical and pathological variables for integration with gene signatures

	Univariate overall survival			Multivariate overall survival		Univariate disease-free survival		
**Variable name**	**p-value**	**Coefficient**		**p-value**	**Coefficient**	**p-value**	**Coefficient**	
ECOG (0[a]/1)	0.14	0.59				0.92	-0.05	
Age	0.001	0.04		9.5E-04	0.05	0.69	0.005	
ACE27 (none[a]/mild/ moderate+severe)	0.02	0.93	1.16			0.28	0.46	-0.06
Sex (male[a]/female)	0.10	0.51				0.64	0.15	
PackYears	0.005	0.02		0.003	0.02	0.32	0.007	
pTNM (1+2[a] vs. 3+4)	0.44	0.24		0.29	0.33	0.39	0.29	
pCompVar (negative[a]/ positive)	7E-06	1.4				7E-05	1.3	

a. Reference category

Supplementary Table 6: Remark criteria checklist

See Supplementary File 5

Supplementary Table 7: Housekeeping gene performance

**Housekeeping gene**	**Correlation coefficient**					**SD[a]**
	**GAPDH**	**GUSB**	**RPL4**	**RPLP0**	**mean-Ct target genes[b]**	
GAPDH	1.00	0.77	0.82	0.83	0.69	1.17
GUSB	0.77	1.00	0.72	0.71	0.77	0.94
RPL4	0.82	0.72	1.00	0.87	0.67	1.05
RPLP0	0.83	0.71	0.87	1.00	0.65	0.98

a. Standard deviation of housekeeping gene across all samples.

b. Correlation of Ct-value of housekeeping gene and average Ct-value of all target genes (n=60).

Supplementary Table 8: Assessment of performance of the gene signature in relevant subgroups by integrated area-under-the-curve (iAUC)

Subgroup	OS			DFS			
	iAUC	95% CI		iAUC	95% CI		n
CompVar: 0	0.71	0.65	0.76	0.65	0.61	0.68	79
CompVar: 1	0.58	0.51	0.65	0.62	0.55	0.70	38
Age<70	0.64	0.59	0.70	0.62	0.57	0.68	88
Age>=70	0.56	0.48	0.66	0.76	0.70	0.82	37
PackYears < median	0.61	0.54	0.69	0.70	0.65	0.75	59
PackYears >= median	0.54	0.48	0.62	0.54	0.47	0.63	66
Female	0.58	0.51	0.67	0.56	0.49	0.63	53
Male	0.63	0.56	0.69	0.64	0.58	0.72	72
pTNM: 1 or 2	0.60	0.52	0.68	0.53	0.45	0.64	43
pTNM: 3 or 4	0.63	0.57	0.69	0.63	0.57	0.70	82
ACE27: 0-1	0.66	0.60	0.73	0.68	0.63	0.75	77
ACE27: 2-3	0.65	0.57	0.74	0.56	0.49	0.63	48
Treatment surgery only	0.51	0.43	0.61	0.69	0.65	0.71	60
Treatment surgery + adjuvant	0.67	0.61	0.73	0.65	0.58	0.73	65

REFERENCES

1. Cancer Genome Atlas Network. Comprehensive genomic characterization of head and neck squamous cell carcinomas. Nature. 2015; 517: 576–82.
2. McShane LM, Altman DG, Sauerbrei W, Taube SE, Gion M, Clark GM. Reporting recommendations for tumor marker prognostic studies. J Clin Oncol. 2005; 23: 9067–72. doi: 10.1200/JCO.2004.01.0454.
3. Smeets SJ, Hesselink AT, Speel EJ, Haesevoets A, Snijders PJ, Pawlita M, Meijer CJ, Braakhuis BJ, Leemans CR, Brakenhoff RH. A novel algorithm for reliable detection of human papillomavirus in paraffin embedded head and neck cancer specimen. Int J Cancer. 2007; 121: 2465–72.
4. van Hooff SR, Leusink FK, Roepman P, Baatenburg de Jong RJ, Speel EJ, van den Brekel MW, van Velthuysen ML, van Diest PJ, van Es RJ, Merkx MA, Kummer JA, Leemans CR, Schuuring E, et al. Validation of a gene expression signature for assessment of lymph node metastasis in oral squamous cell carcinoma. J Clin Oncol. 2012; 30: 4104–10.
5. Johnson WE, Li C, Rabinovic A. Adjusting batch effects in microarray expression data using empirical Bayes methods. Biostatistics. 2007; 8: 118–27. doi: 10.1093/biostatistics/kxj037.
6. Goeman JJ, Geer SA van de, Kort F de, Houwelingen HC van. A global test for groups of genes: testing association with a clinical outcome. Bioinformatics. 2004; 20: 93–9. doi: 10.1093/bioinformatics/btg382.
7. Goeman JJ, Oosting J, Cleton-Jansen AM, Anninga JK, Houwelingen HC van. Testing association of a pathway with survival using gene expression data. Bioinformatics. 2005; 21: 1950–7. doi: 10.1093/bioinformatics/bti267.
8. Benjamini Y, Hochberg Y. Controlling the false discovery rate: a practical and powerful approach to multiple testing. J R Stat Soc Ser B Methodol. 1995; 57: 289–300.
9. Roepman P, Wessels LF, Kettelarij N, Kemmeren P, Miles AJ, Lijnzaad P, Tilanus MG, Koole R, Hordijk GJ, van der Vliet PC, Reinders MJ, Slootweg PJ, Holstege FC. An expression profile for diagnosis of lymph node metastases from primary head and neck squamous cell carcinomas. Nat Genet. 2005; 37: 182–6.
10. Roepman P, Kemmeren P, Wessels LF, Slootweg PJ, Holstege FC. Multiple robust signatures for detecting lymph node metastasis in head and neck cancer. Cancer Res. 2006; 66: 2361–6.
11. Jung AC, Job S, Ledrappier S, Macabre C, Abecassis J, de Reyniès A, Wasylyk B. A poor prognosis subtype of HNSCC is consistently observed across methylome, transcriptome, and miRNome analysis. Clin Cancer Res. 2013; 19: 4174–84.

12. Chung CH, Parker JS, Ely K, Carter J, Yi Y, Murphy BA, Ang KK, El-Naggar AK, Zanation AM, Cmelak AJ, Levy S, Slebos RJ, Yarbrough WG. Gene expression profiles identify epithelial-to-mesenchymal transition and activation of nuclear factor-kappaB signaling as characteristics of a high-risk head and neck squamous cell carcinoma. Cancer Res. 2006; 66: 8210–8.
13. Lohavanichbutr P, Méndez E, Holsinger FC, Rue TC, Zhang Y, Houck J, Upton MP, Futran N, Schwartz SM, Wang P, Chen C. A 13-gene signature prognostic of HPV-negative OSCC: discovery and external validation. Clin Cancer Res. 2013; 19: 1197–203.
14. Rickman DS, Millon R, De Reynies A, Thomas E, Wasylyk C, Muller D, Abecassis J, Wasylyk B. Prediction of future metastasis and molecular characterization of head and neck squamous-cell carcinoma based on transcriptome and genome analysis by microarrays. Oncogene. 2008; 27: 6607–22.
15. Thurlow JK, Peña Murillo CL, Hunter KD, Buffa FM, Patiar S, Betts G, West CM, Harris AL, Parkinson EK, Harrison PR, Ozanne BW, Partridge M, Kalna G. Spectral clustering of microarray data elucidates the roles of microenvironment remodeling and immune responses in survival of head and neck squamous cell carcinoma. J Clin Oncol. 2010; 28: 2881–8.
16. Winter SC, Buffa FM, Silva P, Miller C, Valentine HR, Turley H, Shah KA, Cox GJ, Corbridge RJ, Homer JJ, Musgrove B, Slevin N, Sloan P, et al. Relation of a hypoxia metagene derived from head and neck cancer to prognosis of multiple cancers. Cancer Res. 2007; 67: 3441–9.
17. De Cecco L, Bossi P, Locati L, Canevari S, Licitra L. Comprehensive gene expression meta-analysis of head and neck squamous cell carcinoma microarray data defines a robust survival predictor. Ann Oncol. 2014; 25: 1628–35.
18. Onken MD, Winkler AE, Kanchi KL, Chalivendra V, Law JH, Rickert CG, Kallogjeri D, Judd NP, Dunn GP, Piccirillo JF, Lewis JS, Mardis ER, Uppaluri R. A surprising cross-species conservation in the genomic landscape of mouse and human oral cancer identifies a transcriptional signature predicting metastatic disease. Clin Cancer Res. 2014; 20: 2873–84.
19. Chen C, Méndez E, Houck J, Fan W, Lohavanichbutr P, Doody D, Yueh B, Futran ND, Upton M, Farwell DG, Schwartz SM, Zhao LP. Gene expression profiling identifies genes predictive of oral squamous cell carcinoma. Cancer Epidemiol Biomark Prev. 2008; 17: 2152–62.
20. Batsakis JG. Surgical excision margins: a pathologist's perspective. Adv Anat Pathol. 1999; 6: 140–8.
21. van den Brekel MW, Lodder WL, Stel HV, Bloemena E, Leemans CR, van der Waal I. Observer variation in the histopathologic assessment of extranodal tumor spread in lymph node metastases in the neck. Head Neck. 2012; 34: 840–5. doi: 10.1002/hed.21823.
22. Oken MM, Creech RH, Tormey DC, Horton J, Davis TE, McFadden ET, Carbone PP. Toxicity and response criteria of the Eastern Cooperative Oncology Group. Am J Clin Oncol. 1982; 5: 649–55.
23. Piccirillo JF, Tierney RM, Costas I, Grove L, Spitznagel EL. Prognostic importance of comorbidity in a hospital-based cancer registry. JAMA. 2004; 291: 2441–7. doi: 10.1001/jama.291.20.2441.
24. Schemper M, Smith TL. A note on quantifying follow-up in studies of failure time. Control Clin Trials. 1996; 17: 343–6. doi: 10.1016/0197-2456 00075-X.
25. Heagerty PJ, Lumley T, Pepe MS. Time-dependent ROC curves for censored survival data and a diagnostic marker. Biometrics. 2000; 56: 337–44. doi: 10.1111/j.0006-341X.2000.00337.x.
26. Jiang W, Varma S, Simon R. Calculating confidence intervals for prediction error in microarray classification using resampling. Stat Appl Genet Mol Biol. 2008; 7: Article8. doi: 10.2202/1544-6115.1322.
27. Jiang B, Zhang X, Cai T. Estimating the confidence interval for prediction errors of support vector machine classifiers. J Mach Learn Res. 2008; 9: 521–540.
28. Wahl S, Boulesteix AL, Zierer A, Thorand B, van de Wiel MA. Assessment of predictive performance in incomplete data by combining internal validation and multiple imputation. BMC Med Res Methodol. 2016; 16: 144. doi: 10.1186/s12874-016-0239-7.

SUPPLEMENTARY EXAMPLE R CODE OF THE MULTIVARIABLE GENE SELECTION (LASSO)

```
###########################################################

    #----Example code
    library(glmnet)
    ###########################################################
    #----Simulated some survival data, replace these by real data
    ###########################################################
    P <- 200
    N <- 100
```

```
Train <- matrix(nrow=N,ncol=P)
Train[] <- runif(P*N)
Survival <- rep(c(0,1),each=N/2)
Train[Survival==1,1:5] <- Train[Survival==1,5]+0.5
Time <- runif(N)
Time[Survival==1] <- Time[Survival==1]/2
colnames(Train) <- 1:P
penfac <- rep(1,times=P)
#########################################################
#----Selection with lasso in the form of a LOOCV.
#----Alternatively the lasso can be run repeatedly on the whole data, or on bootstraps.
#----The lasso is likely to select a different set of variables each time it is run on slightly different data or when the cross-validation folds are changed. Especially when the the data are stongly correlated. The aim of running it repeatedly is to stabilise the selection.
#----An unpenalized covariate can be incorporated by setting penfac to 0 for that variable.
#########################################################
N <- length(Survival)     #Number of patients
Selected <- list()   #List for saving selected genes
SavePrediction <- numeric()   #Optionally for saving cross-validated predictions
for(i in 1:N)        #Repeat for each patient
{
  #########################################################
  #Leave one patient out
  Y <- cbind(time=Time[-i],status=Survival[-i])
  X <- Train[-i,]
  #########################################################
  #Fit lasso
  model <- cv.glmnet(X,Y,family="cox",standardize = FALSE,alpha = 1,nfolds=5,penalty.factor=penfac)
  #########################################################
  #Keep track of selected variables
  betas <- model$glmnet.fit$beta[,model$glmnet.fit$lambda==model$lambda.min]
  Selected[[i]] <- names(betas) [which(betas!=0)]
  #########################################################
  #Optionally get an indication of the predictive value
  XTest <- Train[i,,drop=FALSE]
  SavePrediction[i] <- predict(model,newx=XTest,s=c("lambda.min"))
}
#########################################################
#----Add selected variables together and sort/rank
tab <- table(unlist(Selected))
o <- order(tab,decreasing=TRUE)
print(cbind(tab[o]))
#########################################################
#----End of code
```
