# Supplementary material for: Prognostic modeling of oral cancer by gene profiles and clinicopathological co-variables
Source: Oncotarget. 2017 Jul 26;8(35):59312–23. doi: 10.18632/oncotarget.19576 (PMC5601734; doi:10.18632/oncotarget.19576)
Supplement: Supplementary file 4 [file oncotarget-08-59312-s004.docx]

**Supplemental Table 3: Pearson's correlation coefficients and corresponding p-values of technical validation**

| **Gen** | **Correlation coefficient PCR vs Array** | **P-value correlation** |
| --- | --- | --- |
| SPRR2G | -0.98 | 2.30E-13 |
| SERPINB2 | -0.95 | 1.30E-10 |
| MYBPH | -0.94 | 4.90E-10 |
| TNNC1 | -0.94 | 8.10E-10 |
| KRT23 | -0.93 | 1.90E-09 |
| CXCL13 | -0.93 | 4.20E-09 |
| IL8 | -0.93 | 1.50E-08 |
| COL11A1 | -0.9 | 9.10E-08 |
| DEFB103 | -0.89 | 1.30E-07 |
| SYNPO2 | -0.88 | 4.00E-07 |
| PRAME | -0.87 | 5.10E-07 |
| AREG | -0.87 | 7.20E-07 |
| TIMM8B | -0.86 | 1.10E-06 |
| PDHX | -0.86 | 1.50E-06 |
| AMPD1 | -0.86 | 1.50E-06 |
| SPANXA | -0.85 | 1.90E-06 |
| FN1 | -0.85 | 2.40E-06 |
| SPOCK1 | -0.83 | 5.50E-06 |
| EXPH5 | -0.81 | 1.30E-05 |
| KLRC1 | -0.81 | 1.30E-05 |
| SLC5A12 | -0.8 | 2.00E-05 |
| COL5A1 | -0.8 | 2.70E-05 |
| LUM | -0.79 | 2.80E-05 |
| TSPAN11 | -0.76 | 8.50E-05 |
| SELE | -0.76 | 9.60E-05 |
| TPM1 | -0.73 | 2.70E-04 |
| INHBB | -0.7 | 5.80E-04 |
| MPZL2 | -0.7 | 6.40E-04 |
| CLEC3B | -0.69 | 7.40E-04 |
| CCND1 | -0.68 | 9.80E-04 |
| TNXB | -0.67 | 1.10E-03 |
| NDRG1 | -0.67 | 1.30E-03 |
| CTTN | -0.67 | 1.40E-03 |
| ADAM12 | -0.64 | 2.30E-03 |
| SCG5 | -0.64 | 2.40E-03 |
| KIAA1551 | -0.64 | 2.40E-03 |
| P4HA1 | -0.61 | 4.10E-03 |
|  |  |  |
| **Gen** | **Correlation coefficient PCR vs Array** | **P-value Correlation** |
| CALD1 | -0.57 | 8.40E-03 |
| ADCY4 | -0.56 | 9.50E-03 |
| SELP | -0.56 | 1.00E-02 |
| VEGFA | -0.56 | 1.00E-02 |
| COL4A5 | -0.54 | 1.40E-02 |
| KMT2A | -0.53 | 1.70E-02 |
| RGS5 | -0.52 | 1.90E-02 |
| C9orf116 | -0.51 | 2.20E-02 |
| TANC2 | -0.5 | 2.30E-02 |
| PGM5 | -0.48 | 3.30E-02 |
| LRCOL1 | -0.45 | 4.50E-02 |
| COL6A1 | -0.45 | 4.90E-02 |
| PTPRB | -0.44 | 5.00E-02 |
| CCDC88B | -0.42 | 6.30E-02 |
| IRX5 | -0.4 | 8.30E-02 |
| MAGEA12^a^ | -0.37 | 1.10E-01 |
| TPBG^a^ | -0.22 | 3.50E-01 |
| ATP6V0A1^a^ | -0.21 | 3.80E-01 |
| C9orf50^a^ | -0.21 | 4.00E-01 |
| CDK9^a^ | -0.17 | 4.70E-01 |
| SPINK4^a^ | 0.15 | 5.30E-01 |
| MYH10^a^ | -0.07 | 7.50E-01 |
| EIF5^a^ | 0.03 | 8.90E-01 |

a. Genes with correlation coefficients ≤1 SD (mean r=0.64, SD=0.26)
